# Altered RNA editing in 3′ UTR perturbs microRNA-mediated regulation of oncogenes and tumor-suppressors

**DOI:** 10.1038/srep23226

**Published:** 2016-03-16

**Authors:** Liye Zhang, Chih-Sheng Yang, Xaralabos Varelas, Stefano Monti

**Affiliations:** 1 Computational Biomedicine, Boston University School of Medicine, Boston, 02118, MA, US; 2 Department of Biochemistry, Boston University School of Medicine, Boston, 02118, MA, US

## Abstract

RNA editing is a molecular event that alters specific nucleotides in RNA post-transcriptionally. RNA editing has the potential to impact a variety of cellular processes and is implicated in diseases such as cancer. Yet, the precise mechanisms by which RNA editing controls cellular processes are poorly understood. Here, we characterize sequences altered by RNA editing in patient samples from lymphoma, neuroblastoma and head and neck cancers. We show that A-to-I RNA editing sites are highly conserved across samples of the same tissue type and that most editing sites identified in tumors are also detectable in normal tissues. Next, we identify the significant changes in editing levels of known sites between tumor and paired “normal” tissues across 14 cancer types (627 pairs) from The Cancer Genome Atlas project and show that the complexity of RNA editing regulation cannot be captured by the activity of ADAR family genes alone. Our pan-cancer analysis confirms previous results on individual tumor types and suggests that changes of RNA editing levels in coding and 3′UTR regions could be a general mechanism to promote tumor growth. We also propose a model explaining how altered RNA editing levels affect microRNA-mediated post-transcriptional regulation of oncogenes and tumor-suppressors.

RNA editing is a post-transcriptional process that alters nucleotide sequences, and thereby potential function, of virtually all RNA types in eukaryotic cells. Several independently evolved editing mechanisms have been classified, including co- and post-transcriptional base insertions, deletions, substitutions or modifications. RNA editing sites are normally detected by identifying mRNA sequences that do not match the corresponding encoding DNA sequences. Current approaches to RNA editing site detection rely on the comparison of paired RNA-Seq and DNA-Seq data and on the identification of variant sites supported by the former but not the latter. In human, there are two predominant nucleotide substitution types (A to I and C to U) catalyzed by specific enzymes families. The adenosine deaminases acting on RNA (ADARs) catalyze Adenosine (A) deamination in double-stranded RNA to generate inosine (I). As inosine has similar base-paring properties as Guanosine (G), it is read as guanosine by translating ribosomes and reverse transcriptases used to generate next-generation sequencing libraries^1^. Similarly, cytidine deaminase mediates cytidine (C)-to-uridine (U) conversion^2^.

RNA editing can alter protein coding sequences, splicing, transcript stability and expression levels^3^. The malfunction of the RNA editing machinery is associated with tumor growth and, more specifically, significant changes in editing levels at specific sites have been associated with cancer^4^,^5^,^6^. For example, the hypo-editing of A-to-I sites were observed in multiple human cancers^7^, and the increased editing level in the coding sequence of *AZIN1* mRNA has been shown to stabilize the corresponding protein and to promote cancer proliferation^8^.

Despite this growing evidence, few transcriptome-wide studies have explored RNA editing events in cancer. In this work, we first evaluated whether a significant number of novel (potentially somatic) RNA editing sites appeared in cancer samples. We leveraged publicly available parallel sequencing datasets (with both DNA-Seq and RNA-Seq data) to characterize RNA editing in cancer samples, and selected neuroblastoma (NB), Diffuse large cell lymphoma (DLBCL) and Head and Neck Squamous cell Carcinoma (HNSC) to cover tumors with various mutation rates and hematological and solid tumors^9^,^10^. We demonstrate that the A-I RNA editing sites identified in cancer are neither sample-specific nor somatic (i.e., present in cancer but not in normal cells), but commonly shared by the majority of samples of the same tissue, including the paired pathologically normal adjacent tissues.

Given the lack of evidence for the presence of cancer-specific (somatic) RNA editing sites, we next focused on those sites that are significantly altered between tumors and paired normal samples. For this analysis, we focused on known RNA editing sites annotated in the Rigorously Annotated Database of A-to-I RNA Editing (RADAR), which is the most up to date RNA editing sites database^11^. Most previous studies focused on one particular tissue type and studied few RNA editing sites with low-throughput assays. Furthermore, there is still debate about the functional role of RNA editing in cancer^12^,^13^. Two pan-cancer RNA editing studies were just published^14^,^15^, both of which focused on showing the clinical relevance of RNA editing in cancer. However, it is still largely unclear how these changes are regulated and how they promote tumor progression. With these considerations in mind, we carried out a pan-cancer analysis of the cancer genome atlas (TCGA) datasets corresponding to 14 tumor types and 627 pairs of tumor/normal samples, to identify which of the known RNA editing sites manifest significant editing level changes between tumors and paired normal samples, to predict the functional consequences of these changes, and to assess their potential contribution to tumorigenesis.

## Results

### RNA Alignment Artifact Removal (RAAR): Python pipeline to obtain confident variant calls from RNA-Seq

**Figure 1:**
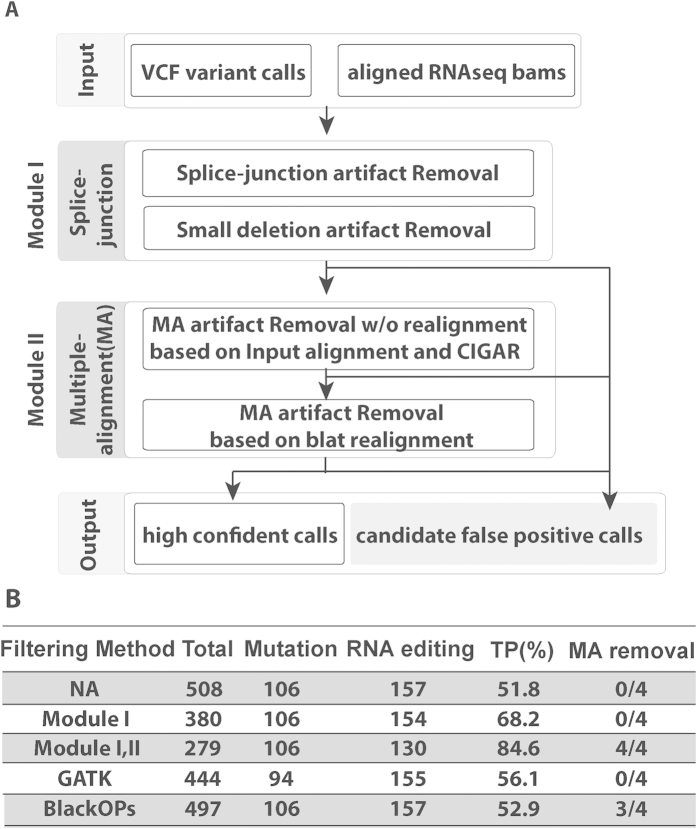
RAAR pipeline flow and performance comparison. (**A**) RAAR pipeline flowchart. (**B**) Column ‘Mutation’ reports the number of variant calls supported by GM12878 DNA-Seq data; Column ‘RNA editing’ reports the number of variant calls annotated as RNA editing sites in the RADAR database^11^. True Positive (TP) rate is defined as 100 * (Mutation + RNA editing)/Total. Multiple Alignments (MA) removal of 4 known cases^52^ is shown in the last column.

Previous studies suggest that multiple sources of artifacts contribute to false positive variant calls from RNA-Seq data, such as splice junction-related artifacts (the misalignment of RNA-Seq reads around exon splice-junctions often leads to false positive calls), and multiple alignment-related artifacts (arising from incorrect alignment of reads to highly homologous/repetitive regions in the genome)^16^,^17^. To overcome these problems, we developed an RNA-Seq based Alignment Artifact Removal (RAAR) pipeline (https://github.com/bioliyezhang/RAAR) to eliminate these potential sequencing artifacts (see Fig. 1A and supplemental Materials for details). Our method is applicable to both RNA editing events and DNA mutation calls detected from RNA-Seq data.

The main advantages of our pipeline are: 1) *Improved Accuracy*. We optimized our algorithms to select the best alignment out of *Blat* realignment output. We compared our method with the GATK realignment method^18^ to remove small indel related alignment artifacts on chr21 of the GM12878 dataset^19^. Our method achieved a significantly higher True Positive rate (84.6%) than the *GATK* realigned output (56.1%) (Fig. 1B). We also compared our method with *BlackOPs* to remove multiple alignments artifacts^20^. Our method removed all four known multiple alignment artifacts, while *BlackOPs* can remove three out of the four known cases; 2) *No reliance on genome annotation*. Our pipeline works without genome annotation, while most existing tools depend on exon-intron annotations^16^,^21^. However, if available, our pipeline can use exon-intron boundary annotations to improve accuracy in removing splice junction-related artifacts; 3) *Easy to use*. We adopted the most commonly used VCF and BAM formats as the input/output of the pipeline; we also aimed to simplify installation by making the tool dependent only on commonly available tools such as *samtools*, *bedtools* and *Blat*.

### The majority of RNA editing sites in cancer genomes are canonical A-I RNA editing sites

To characterize RNA editing sites in 13 DLBCL samples^22^, 10 NB samples^10^ and 14 HSNC samples^23^, we applied VarScan2 to call variants from RNA-Seq using a p-value cutoff of 0.01^24^. We next removed the DNA mutation variants that were supported by the paired DNA-Seq data, and other artifacts using the RAAR pipeline described above.

After filtering, a given base substitution at a given genomic location is designated as an RNA editing *site* if there is at least one sample with a minimum of 5 RNA editing supporting reads covering the site, and if at least 5% of all reads manifests the substitution (the thresholds were adopted from recommendations in VarScan 2^24^). We define the percentage of reads supporting a given RNA editing nucleotide substitution as its editing *level*. Finally, we define the editing of any one RNA editing site in any one sample as a single RNA editing *event*.

**Figure 2:**
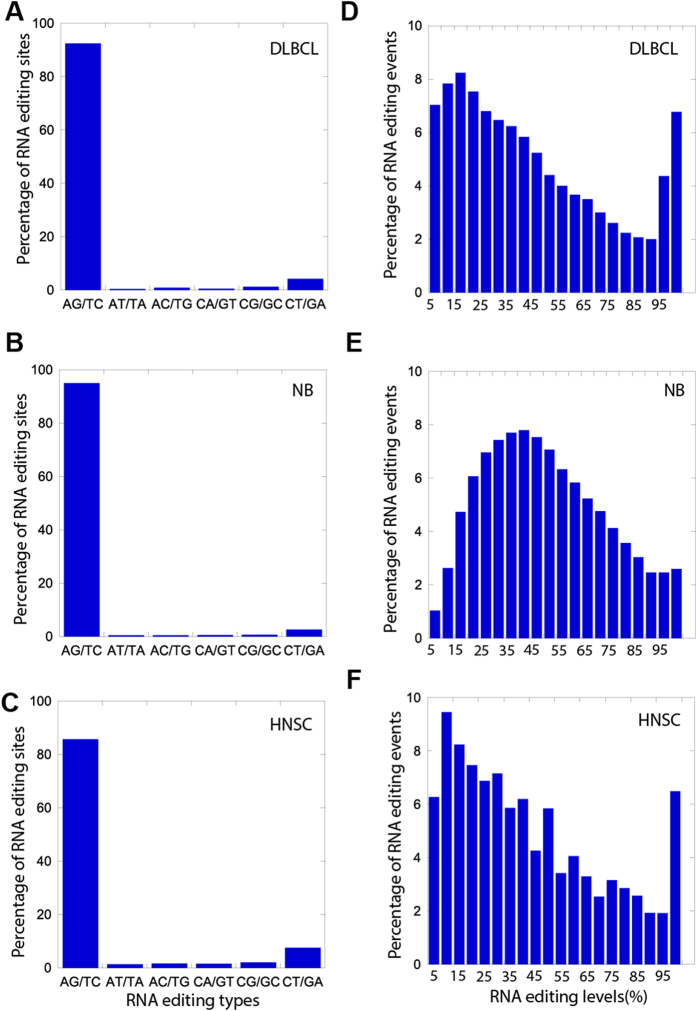
RNA editing sites identified in cancer shared similar features to RNA editing sites in normal samples from previous studies. (**A**–**C**) Frequency distribution plots of nucleotide substitution types from pooled RNA editing events in three types of cancer. (**D**–**F**) Frequency distribution plots of editing levels from pooled RNA editing sites in three types of cancer. 5 bp bin size and 3 moving average was used to smooth the data.

Based on these criteria, we obtained a list of RNA editing events for each sample. The large variation in the number of RNA editing events is associated with the variable sequencing depth among samples (Table S1). To assess global RNA editing patterns, we pooled the RNA editing events from all samples of the same cancer type, and measured the frequency of nucleotide substitution types (Fig. 2A–C), as well as the distribution of editing levels across all events (Fig. 2D–F). A-I (A-G) and C-U (C-T) substitutions accounted for >95% of the RNA editing events in all three cancers. Furthermore, consistent with previous reports, the majority of RNA editing sites in lymphoma was edited at a low level (~15%)^16^ (Fig. 2D). Interestingly, the editing level in neuroblastoma peaked at ~35–40% (Fig. 2E), which is significantly higher than the one observed in DLBCL and HNSC. To ascertain that the editing level patterns observed in Fig. 2D–F recapitulate the patterns in individual samples, we generated separate plots for each of the samples with sufficient sequencing depth. The corresponding patterns closely matched those obtained from the pooled data, with the higher editing level in NB also observed in the single samples’ comparison (Fig. S1–3). We next tested whether the expression level of ADAR family genes could explain the pattern differences between tumor types. Of notice, the higher editing level in NB was associated with a significantly higher expression of ADAR family genes, *ADARB1* and *ADARB2* (Fig. S4).

### The majority of A-I RNA editing sites are modified in *all* samples

**Figure 3:**
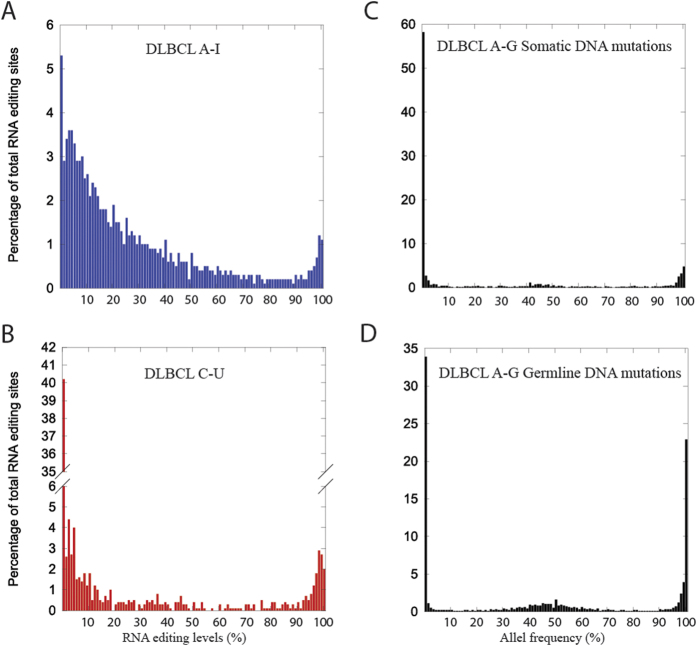
The majority of A-I RNA sites are edited at low level. Frequency distribution plot of editing levels from pooled A-I RNA editing events (**A**) C-U RNA editing sites (**B**), A-G type somatic mutations (**C**) and A-G type germline mutations (**D**) across all samples in DLBCL after retrieving events with relaxed threshold.

Due to the initial stringent threshold to obtain a high-confidence set of RNA editing sites, we re-examined these RNA editing sites across all samples in Figs 3A and S6A. Unmodified sequences (0%-level editing) represented a very low proportion of all possible A-I RNA editing events (~5%), suggesting that A-I RNA editing events could be reliably detected in most samples. The equivalent analysis of C-U editing events (Figs 3B and S6B) yielded a much larger portion (~40%) of predicted 0%-level C-U editing events, suggesting there are many more unedited C-U sites than unedited A-I sites (~40% vs ~5%). Furthermore, to eliminate a potential sequencing technology bias unique to the A-G (I) nucleotide substitution, we analyzed the allele frequency distribution of DNA A-G variants as controls (Figs 3C,D and S6C,D). Across all samples, >50% somatic and >30% germline events had zero “edited” allele frequency, ruling out the possibility of an A-G substitution type sequencing bias.

The observed editing patterns support the hypothesis that the A-I RNA editing sites are modified in almost all samples. To test this hypothesis, we selected the sites with sufficient coverage (≥20X coverage) in all samples. For each of these sites, we counted the number of samples showing presence (non-zero level) of editing. The resulting distributions are displayed in Fig. S7A–C (DLBCL, NB, HNSC), and show that most of the editing sites (~60%) are observed in all samples, with less than 1% sites observed in fewer than 3 samples. Furthermore, the editing sites not detected in all samples are enriched for low-level editing events (Fig. S7D–F), thus suggesting that a low sampling fraction and random sampling in RNA-Seq might be the reason for the events to go undetected^25^.

### Cancer cells do not generate cancer-specific A-I RNA editing sites

We wanted to ascertain whether RNA editing could function as an additional mechanism contributing to malignant transformation by generating novel (i.e., somatic) A-I RNA editing sites that are unique to cancer samples. As the majority of A-I RNA editing sites could be seen in all samples, this seemed unlikely.

**Figure 4:**
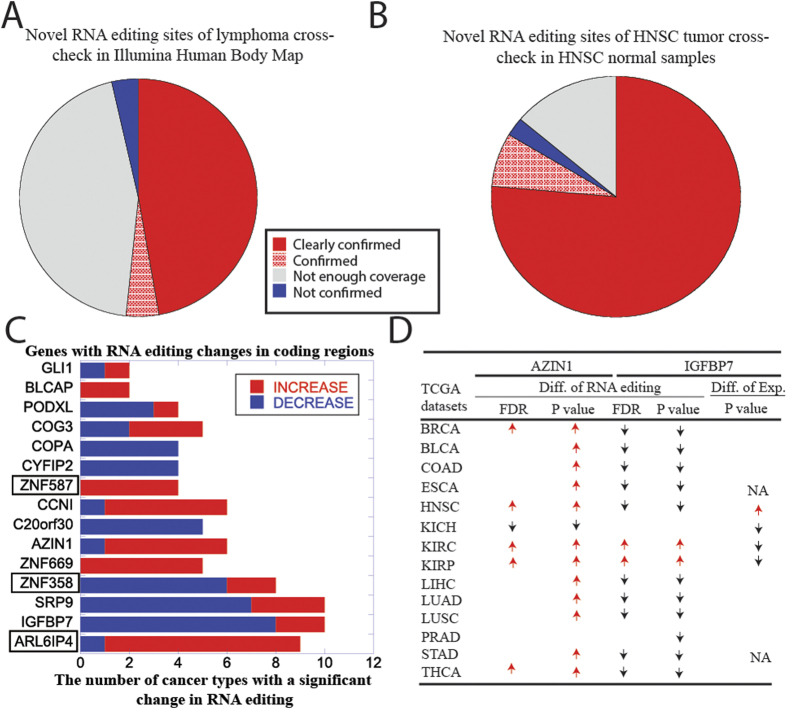
Altered RNA editing in coding region is common across multiple cancer type. (**A**) Novel A-I RNA editing sites identified in DLBCL were tested for inclusion in the Illumina human body map (n = 16). Sites colored in red were supported by two or more samples, sites marked by red dots were supported by one sample, sites colored in gray had no sufficient coverage in any sample, and sites colored in blue were not supported by any sample with sufficient coverage. (**B**) For the novel A-I RNA editing sites identified in HNSC, we examined their occurrence in normal samples from HNSC patients (including but not restricted to their paired normal samples). (**C**) Stacked plot counting, for each gene, the number of tumor types showing increased (red) or decreased (blue) editing levels. Genes framed by rectangles were not previously reported as differentially edited. (**D**) Table showing, for each cancer type, whether there was a increased editing (↑) in *AZIN1* and a decreased editing (↓) in *IGFBP7* between tumor and normal samples based on FDR and unadjusted p value. The differential expression of *IGFBP7* mRNA between tumor and paired normal samples was also assessed by a student’s t-test.

Since RNA-Seq data from paired normal samples for DLBCL and NB were unavailable, we could not address this question directly in these tissues. However, based on the assumption that cancer specific RNA editing should not be detected in normal cells, we checked for the presence of the newly discovered RNA editing sites in the Illumina Human Body Map (HBM) data 2.0^26^. As shown in Figs 4A and S8A, the majority of sites with sufficient coverage were also found in the HBM data (non-grey color region), and those that were not found had a much lower read coverage (Fig. S8B,C), suggesting that the combined effect of insufficient sequencing coverage and low editing levels likely explains their non-detection.

Thanks to the availability of paired normal samples in the HNSC dataset, we next examined whether the novel RNA editing sites identified in the tumors could also be observed in the paired normal tissues. As shown in Fig. 4B, a clear majority of RNA editing sites detected in tumors could also be observed in the paired normal, with lack of detection likely attributable to low coverage (Fig. S8D). In summary, our analysis provides no evidence for the existence of somatic, i.e., cancer-specific, RNA editing sites.

### Pan-cancer analysis of coding region confirmed known altered RNA editing sites

Given the lack of evidence for the presence of somatic A-I RNA editing sites, we focused on the significant editing level changes between normal and tumor samples of known RNA editing sites included in the RADAR database^11^. We processed all TCGA datasets (Table 1) with a sufficient number (≥10) of paired normal samples, and performed paired Student’s t tests with multiple hypotheses correction to identify significantly changed RNA editing sites in the coding region.

Our analysis identified a total of 166 differentially edited genes (the most frequent ones are shown in Fig. 4C), including a majority of the previously reported genes (the ones not enclosed in rectangles in Fig. 4C), and 153 novel genes, with a median of 27 differentially edited genes per tumor type, from a minimum of 11 in ESCA to 62 in BRCA (Table S3).

We then further analyzed the editing sites in *AZIN1* and *IGFBP7*, whose functional role and upstream regulation were previously reported^8^,^27^. While only five cancer types showed a significant increase in *AZIN1* RNA editing levels with FDR ≤0.05, most cancer types (12 out of 14) showed increased editing with nominal P-value ≤0.05 (Fig. 4D). Furthermore, although it is known that *ADAR* is responsible for *AZIN1* editing^8^, we did not observe *ADAR* overexpression in any of these tumor types. In contrast, two of the five cancer types with significantly increased *AZIN1* editing showed a significantly decreased expression of *ADAR* in tumor samples (Fig. S9). Consistent with previous results, we observed a dominant pattern of decreased RNA editing in *IGFBP7*^27^. A previous study in skin cancer suggested that decreased RNA editing levels of *IGFBP7* results from failure to edit excess amount of highly expressed *IGFBP7* transcripts in tumors; however, in our analysis only HNSC showed a significant increase in *IGFBP7* total mRNA levels (P-value ≤0.05) (Fig. S10).

### 3′UTR RNA editing sites of the same gene may be regulated differently

RNA editing sites in the protein coding regions only account for a small proportion of total RNA editing sites in the genome. The majority of RNA editing sites resides at intronic region, and several of these intronic sites can affect RNA splicing to regulate the functions of genes^28^,^29^,^30^. However, due to the large variation in intronic read coverage in RNA-Seq, we were unable to study the intronic RNA editing sites in a systematic manner. On the other hand, there is an enrichment of RNA editing sites in the 3′ UTR^31^, where microRNAs bind. Previous studies have suggested that RNA editing of microRNA target sites in 3′ UTRs may prevent the post-transcriptional repression by microRNAs^32^. To address this question, we examined all RNA editing sites reported in the RADAR database, and identified all those differentially edited in the 3′ UTR. We identified more than 2000 genes with at least one site differentially edited between tumor and normal in at least one tumor type.

Unlike the coding regions, 3′ UTR regions can contain from a few to tens of RNA editing sites. We thus investigated whether all the RNA editing sites of the same gene were concordantly regulated (i.e., all sites increased or all sites decreased their editing level in tumors compared to normals). We first focused our attention on cancer census genes listed in the COSMIC database^33^ and with at least two differentially edited sites (n = 9). Of these, 7 genes showed a consistent direction of editing level changes in all their significantly changed sites, while both directions of change were observed in the editing sites of *MDM4* and *CASP8* (Table 2). Similar patterns were observed when all genes were included in the analysis (Table S4). Taken together, these results suggest that the regulation of editing levels of the multiple RNA editing sites within a gene is not always concordant, with different sites of a transcript regulated differently, sometimes in opposite directions.

### Increased RNA editing on 3′ UTR region of *MDM2* may increase its mRNA levels by abolishing microRNA-mediated repression

Given the abundance of RNA editing sites in 3′ UTR and the important role microRNAs play in tumor suppression^34^, it is crucial to understand whether altering RNA editing levels in 3′ UTR can contribute to escaping microRNA repression and promoting tumorigenesis. To this end, association between altered RNA editing levels and protein levels should ideally be measured, given the imperfect matching between microRNAs and mRNA’s 3′ UTR sequences^35^. However, paired proteomic data for the samples analyzed were not available. Given the mRNA destabilization mechanism by microRNA^36^, we argue that it is still possible to detect the association between RNA editing levels and mRNA level changes.

**Figure 5:**
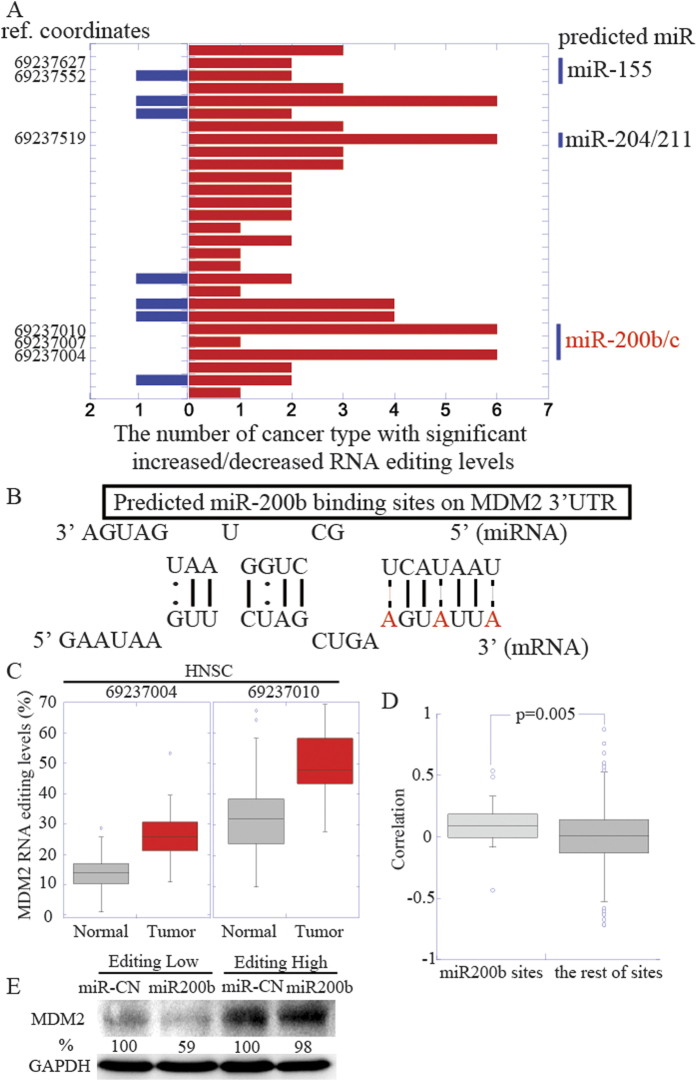
Increased RNA editing in the 3′ UTR of *MDM2* abolishes microRNA repression. (**A**) Number of tumor types showing significant increased (red) or decreased (blue) editing levels for each RNA editing site in the *MDM2* 3′ UTR region. (**B**) The more detailed binding pattern is predicted using http://microrna.osumc.edu/mireditar, with RNA editing sites in red. The solid lines represent the perfect base-pairing between microRNA and mRNA, the dashed lines represent the base-pairing that can be disrupted by RNA editing, and the dotted lines represent the G-U wobble base-pairing^53^. (**C**) Boxplot of RNA editing levels at coordinates 69237004 and 69237010. (**D**) Pearson correlation between changed mRNA editing levels and changed mRNA levels was calculated for each site across all cancer types. The Student’s t-test was performed to compare the correlations of three hsa-miR-200b target sites with the rest of the RNA editing sites in the 3′UTR of *MDM2*. (**E**) *MDM2* levels upon the overexpression of microRNAs by Western blot. Relative levels of *MDM2* abundance were calculated in reference to control gene GAPDH and then normalized to the control microRNA in each cell line.

We focused our analysis on genes known to regulate tumor progression. In particular, we selected cancer census genes that undergo significant changes in RNA editing levels *and* whose expression is known to be repressed by microRNAs. We further restricted our analysis to those genes that showed consistent editing level increase/decrease across the entire 3′ UTR. The resulting top ranking gene was *MDM2*, a key oncogene in the p53 pathway with elevated expression in multiple tumor types^37^, and 11 out of 14 tumor types showed altered RNA editing at the 3′ UTR of MDM2, with a median of 5 sites per tumor type. Several microRNAs are reported to repress *MDM2* expression levels. To evaluate whether RNA editing may regulate *MDM2*, we first identified the microRNAs predicted to bind at RNA editing sites with significant editing level changes in at least one tumor type. We found three microRNAs matching these criteria (Fig. 5A). Of these, only hsa-miR-200 b/c was previously shown to be negatively associated with *MDM2* expression^38^. Moreover, our analyses suggested that the target sites of hsa-miR-200 b/c were among the most commonly altered RNA editing sites across multiple cancer types (Fig. 5A). We next visualized the predicted binding between microRNA and *MDM2* (Fig. 5B). Interestingly, both start and end of the seed sequences were converted to I(G) by RNA editing, suggesting that editing in these three sites are very likely to prevent the binding of microRNA. Two out of three sites showed significantly increased RNA editing levels in several cancer types, with the remaining site showing significant increase in HNSC (Fig. 5A,C). Since multiple factors are known to regulate *MDM2* expression, with the regulation by any one microRNA likely accounting for only a small portion of total regulation, we would expect to see a positive, but not necessarily strong, correlation between the editing level of these sites and *MDM2* mRNA level. We thus computed the correlation score between changed *MDM2* expression level and changed *MDM2* RNA editing levels for each site in each tumor type. A comparison of the correlation distributions within the three hsa-miR-200 b/c sites and in the remaining (non hsa-miR-200 b/c) sites showed a significantly higher positive correlation in the former (Fig. 5D, p-value: 0.005). The median correlation score for these three sites is around 0.1, while the median correlation score for the other sites is around zero. To further validate the disruption of microRNA-mediated repression by RNA editing, we selected one cancer cell line with high RNA editing level (BT474) and one cell line (MDA-MB-231) with low RNA editing level at the 3′ UTR region of *MDM2* (Table S5). We hypothesized that the cell line with higher RNA editing levels would be less affected by hsa-mir-200b. We therefore transfected each cell line with control microRNA and hsa-mir-200b mimics. Confirming our hypothesis, *MDM2* protein level in the cell line with low editing level dropped to ~60%, while MDM2 protein level in the cell line with high editing level was not affected (Fig. 5E). This example provides a proof of principle of how increased RNA editing in cancer samples can upregulate proto-oncogene activity and thus promote tumor progression.

### The direction of RNA editing level changes is cancer specific

**Figure 6:**
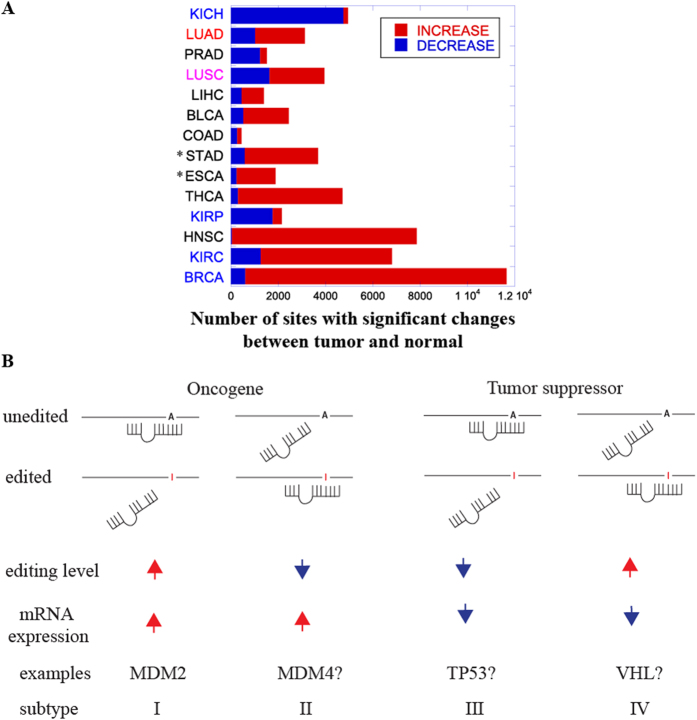
Summary of altered RNA editing sites in coding and 3′ UTR regions across multiple cancer types and A model of tumor progression mediated by altered RNA editing levels on 3′ UTR. (**A**) Stacked bar plot counting, for each cancer type, the number of RNA editing sites (including coding region and 3′ UTR) with significantly increased (red) or decreased (blue) editing levels. Each tumor type is color-coded. Black denotes no changes in the expression of ADAR genes between tumor and normal samples. Blue and red indicate that ADAR family genes have decreased and increased expression in the tumor samples, respectively. Purple indicates that *ADAR* and *ADARB1* show opposite directions of expression changes. Tumor types marked by a (*) denote tumor types for which normalized expression data was not available. (**B**) An expanded model of how altered RNA editing levels on 3′ UTR can up-regulate oncogenes and down-regulate tumor suppressors by interfering with their modulation by microRNAs. In subtypes I and III, RNA editing inhibits the binding of microRNA, while in subtypes II and IV, RNA editing creates novel microRNA binding sites. In the (**A**) represents the original unedited nucleotide, while I represents the edited nucleotide. The red upward arrow denotes up-regulation, while the blue downward arrow denotes down-regulation.

Both increased and decreased RNA editing of mRNAs have been previously shown to be associated with tumor progression^7^,^8^. Brain, prostate, large cell carcinoma of the lung, kidney renal cell carcinoma (KIRP), and testis tumor all undergo a global decrease in RNA editing levels^7^, while increased RNA editing is observed in liver, breast, leukemia and esophageal cancer^8^,^39^. Our pan-cancer, genome-wide analysis on breast, liver, esophageal and prostate cancer confirmed previous results: we observed hypo-editing in prostate (PRAD), KICH and KIRP, and hyper-editing in liver (LIHC), BRCA and ESCA (Fig. 6A and Table S6). These reproducible results suggest that hyper- or hypo-editing in a given tumor type is not random and may provide selective advantage. In addition, our results suggested that the regulation of RNA editing in cancer subtypes from the same tissue can be different: under-editing events were more frequent than over-editing events in KIRP and KICH, while over-editing events were more frequent in KIRC. Finally, in contrast to previous findings that the level of RNA editing decreases in lung cancers, we found that both LUAD and LUSC exhibited dominant over-editing events. These results were reproduced in a recent pan-cancer study^15^ and were consistent with recent findings that the overexpression of ADAR enhances the tumorigenesis in human lung tissue^40^.

## Discussion

Our analysis uncovered a distinct feature of A-I RNA editing sites: they are high-recurrence events shared by most samples. This unique feature of RNA editing sites is in marked contrast with somatic DNA mutations. While the recurrence of editing sites across individuals was previously reported^41^, our analysis expand upon those results by showing that A-I RNA editing sites are shared by *most* samples from the same tissue.

Limited overlap of RNA editing sites was previously observed between different cell lines and datasets^16^,^42^. However, those results heavily depended on the chosen detection threshold, and they are likely to have under-counted the lowly edited sites due to too stringent a threshold. Here we have shown that a relaxed threshold recovers low edited sites (Compare Fig. 2D–F and Figs 3A and S6A), consistent with previous observations of a prevalence of lowly edited (<1%) A-I RNA editing sites^43^. We argue that a two-step detection procedure — whereby i) a stringent cutoff is used to detect RNA editing sites; and ii) the occurrence of RNA editing events (within the detected sites) in the whole population is re-assessed based on a less stringent threshold — will show that A-I RNA editing sites are shared by most samples.

While the RNA editing sites in coding regions directly alter protein sequences and functions, the RNA editing in 3′ UTR may regulate the binding of microRNAs. MicroRNAs play important roles in tumorigenesis, and the deregulation of microRNA-mediated repression is likely to contribute to cancer^34^,^44^. Therefore, we identified frequently changed 3′ UTR RNA editing sites and assessed whether they may promote tumor progression. We showed positive correlation between changes of RNA editing levels and *MDM2* mRNA expression (Fig. 5). The median correlation across multiple tumor types, while significant, was not very high, likely reflecting the multiple layers of regulation controlling this key gene^37^. The negative correlation previously reported between *MDM2* and hsa-miR-200 b/c results from repression of hsa-miR-200bc expression by *MDM2*^38^. We hypothesize that hsa-miR-200bc may be able to repress *MDM2* expression as well, suggesting a negative feedback loop between the two. Given the widespread presence of A-I RNA editing sites and microRNA binding sites in the 3′ UTR, RNA editing-regulated microRNA binding might be a common mechanism to control genes expression, and may also play a role in physiological, non-disease conditions.

Although mechanistic models have been proposed to explain how editing changes in the coding region of genes may occur^8^,^27^, these models failed to adequately explain some of the patterns we observed in multiple cancer types. For instance, the increased RNA editing of *AZIN1* could not be explained by increased *ADAR* expression (Figs 4D and S9). Similarly, we did not observe increased mRNA of *IGFBP7*, which would be necessary to explain the decreased editing of *IGFBP7* (Fig. 4D). Additionally, we showed that the editing regulation is not at the gene/transcript level, but at the sub-transcript level: different sites on the same mRNA can be regulated differently (increased editing levels for some, decreased editing levels for others) (Table S4). This is consistent with the processing of *ADAR*, which is processed in a region-by-region manner rather than in a sequential manner like RNA polymerase. This region-specific regulation is also consistent with the regulation by RNA binding proteins. Finally, we showed that the relative proportion of sites with increased *vs*. decreased editing levels could be partially explained by the differential expression of three RNA binding proteins – *RPS14*, *SRSF9* and *DHX15* – in a subset of the cancer types we analyzed (Supplementary Results). However, as there are 692 mRNA binding proteins in the human genome^45^, it is likely that additional RNA binding proteins can regulate RNA editing events.

Our result on *MDM2* regulation is consistent with the previously proposed model that “RNA editing events may provide an additional layer of control of miRNA-mediated repression”^32^. Because the model only considers cases where RNA editing events abolish existing microRNA binding events and repression, we would predict that, to facilitate tumor progression, RNA editing decreases in oncogenes to upregulate their expression/translation/stability and increases in tumor suppressors to downregulate their expression/translation/stability. However, we did observe cases where RNA editing increases in oncogenes and decreases in tumor repressor genes, which cannot be fully explained by the proposed model.

Therefore, we propose an expanded model of how both increased *and* decreased RNA editing can promote tumor growth (Fig. 6B). The previous model fits with subtype I in Fig. 6B: microRNA mediated repression of oncogenes is abolished by increased RNA editing levels, thus leading to the overexpression of oncogenes and tumor progression. On the other hand, *TP53* fits with subtype III: decreased RNA editing levels in tumor repressors downregulate their expression, as the less edited *TP53* mRNA can be subjected to microRNA mediated repression (Table S7). Consistent with the prediction of our model, multiple tumor types show a decreased *TP53* mRNA editing and expression in tumors, although none of them with an FDR below 0.05. Conversely, only HNSC showed increased editing levels in two 3′ UTR RNA editing sites, and a corresponding significant increased *TP53* mRNA expression in tumors (P-value 0.008) (Fig. S12). When we expanded the analysis to identify subtype III RNA editing changes in the compiled list of tumor suppressor genes (http://bioinfo.mc.vanderbilt.edu/TSGene/), we found 5 additional RNA editing sites whose levels are positively correlated with corresponding mRNA levels in at least one cancer type (Table S8). While microRNA binding is lost upon RNA editing in subtypes I and III, a novel microRNA binding site can be created in subtypes II and IV. It is more difficult to locate potential functional RNA editing sites that create novel miRNA binding sites, as they are not annotated in the existing databases. However, the tumor suppressor gene *VHL* and the oncogene *MDM4* seem to show patterns consistent with subtypes II and IV (Table 2). Taken together, these results suggest that our proposed expanded model of miR-mediated mRNA regulation provides a general framework to understand how different types of RNA editing changes can promote tumor growth.

In summary, our RNA editing profiling of three tumor types found no evidence of cancer specific or novel RNA editing events. On the other hand, our pan-cancer analysis of TCGA datasets corresponding to 14 cancer types identified RNA editing sites recurrently altered in coding and 3′ UTR that may facilitate tumor progression, and predicted the potential regulation mechanisms for many of such alterations. These results can serve as a starting point to further understand the mechanisms of RNA editing regulation and the functional roles of RNA editing sites that significantly change in tumors vs. normals.

## Material and Methods

### Data collections

The dbGaP accession numbers of the accessed datasets are: phs000235.v2.p1 for the lymphoma dataset^22^; phs000467 for the neuroblastoma dataset^10^; and phs000178.v8.p7 for the TCGA datasets. The TCGA BAM files aligned to hg19 reference were downloaded from CGHub (https://cghub.ucsc.edu/) using the GeneTorrent application 23. The accession number of RNA-Seq data from the illumina human body map 2.0 is GEO30611^26^.

### RNA editing site calling and filtering

The adaptor trimming was performed on the FASTQ files from the illumina human body map by using *Trim-galore* (version0.2.8, http://www.bioinformatics.babraham.ac.uk/projects/trim_galore). The RNA-Seq reads were aligned to hg19 reference genome using *Tophat* v2.0.4^46^. The optical and PCR duplicates were removed using *samtools* v0.1.18 *rmdup* function^47^. An *mpileup* file was generated by samtools for each sample and used to call variants using *Varscan* 2.2.11^24^. A series of quality filters was then applied to minimize the false positive rate (Table S2). Next we filtered out DNA mutation calls by examining the paired DNA-Seq data, the variant would be removed if their allele frequency in paired DNA-Seq is ≥5%. Finally, we applied our own pipeline to remove multiple types of alignment artifacts (Fig. 1 and details in supplementary material).

When retrieving the lowly edited and low coverage RNA editing events based on a relaxed threshold for the previously identified highly confident sites, we discarded any event with a total coverage lower than 10 reads.

### The validation of novel RNA editing sites in HBM and normal samples of TCGA HNSC

To validate the novel RNA editing sites we identified in DLBCL, we calculated the read depth and editing levels for each of these sites in the HBM dataset (n = 16), and for each sample we excluded those sites with read depth below 10 reads. When the read depth was ≥10, the number of RNA editing supporting reads was ≥1 and the allele frequency was ≥1%, we considered the sample as supporting the RNA editing event. When an RNA editing event was detected in more than two samples, we considered the corresponding site a real RNA editing site based on the HBM dataset. When the RNA editing event was detected in only one sample, we considered this site likely to be a real RNA editing site. When no sample met the minimum read coverage threshold, we concluded we did not have enough information to make a call on this site. When at least one sample had sufficient coverage but no sample supported the RNA editing event, we considered this site not to be an RNA editing sites based on HBM. To validate the potential cancer-specific RNA editing sites in HNSC samples in their paired normal tissue samples, we adopted the same criteria.

### The prediction of regulatory microRNAs whose binding is affected by RNA editing

We downloaded the predicted microRNA binding data of highly conserved microRNA families from http://mircode.org/info.php^48^. We used intersectBed of BedTools to find RNA editing sites that overlap with microRNA seed regions^49^. We then used *miR-EdiTar* to visualize the binding between a microRNA and its target 3′ UTR sequences^50^.

### Identification of RNA editing sites whose levels change significantly between tumor and normals

For all the tumor-normal pairs in 14 TCGA datasets, we calculated the RNA editing level (the percentage of “edited” allele frequency) for each coding and 3′ UTR RNA editing sites in RADAR database. Then we used the paired student’s t test to evaluate whether tumor samples showed a significantly increased or decreased editing level compared with normal samples for each site within individual tumor type. The FDR (B-H) cutoff of 0.05 was adopted to control for multiple hypothesis testing.

### Correlation analysis between the changes of mRNA levels and RNA editing levels

As copy number gains in *MDM2* are frequently observed in tumors and can confound our correlation analysis, we first removed the tumor samples where a copy number alteration is detected by GISTIC2. We then measured the association between changed log_2_ *MDM2* expression level and changed *MDM2* RNA editing levels by Pearson correlation for each site in each tumor type. We performed the Student’s t-test to compare of the correlation distributions within the three hsa-miR-200b sites and in the remaining (non hsa-miR-200b) sites.

### Cell culture, plasmids, and transfections

Human breast cancer lines MDA-MB-231 and BT474 cells were purchased from ATCC, and grown at 37 °C in DMEM and RPMI 1640 media. Media were supplemented with 10% FBS and 1X pennicillin/streptomycin. Cells were transfected with 50 nM control microRNA or miR200 by Lipofectamine RNAiMAX reagents (Invitrogen, Grand Island, NY, USA).

### Immunoblotting

Lysate was collected 72 h after transaction, and analyzed by immunoblotting using anti-MEM2 (Santa Cruz, sc-965) and anti-GAPDH (Genescript, A00192-100) as previously described^51^.

## Additional Information

**How to cite this article**: Zhang, L. *et al.* Altered RNA editing in 3′ UTR perturbs microRNA-mediated regulation of oncogenes and tumor-suppressors. *Sci. Rep.* **6**, 23226; doi: 10.1038/srep23226 (2016).

## Supplementary Material

Supplementary Information

Supplementary Dataset 1

Supplementary Dataset 2

Supplementary Dataset 3

## Figures and Tables

**Table 1:** summary of TCGA tumor dataset analyzed.

TCGA Type	TCGA Abbreviation	Number of Sample Pairs	Normalized expression availability
Breast invasive carcinoma	BRCA	99	Available
Bladder Urothelial carcinoma	BLCA	19	Available
Colon adenocarcinoma	COAD	49	Available
Esophageal adenocarcinoma	ESCA	13	NA
Head and neck squamous cell carcinoma	HNSC	43	Available
Kidney Chromophobe	KICH	25	Available
Kidney renal clear cell carcinoma	KIRC	72	Available
Kidney renal papillary cell carcinoma	KIRP	32	Available
Liver hepatocellular carcinoma	LIHC	49	Available
Lung adenocarcinoma	LUAD	43	Available
Lung squamous cell carcinoma	LUSC	51	Available
Prostate adenocarcinoma	PRAD	51	Available
Stomach adenocarcinoma	STAD	34	NA
Thyroid carcinoma	THCA	57	Available
Total		627	12

**Table 2:** Summary of 3′ UTR editing change directions in COSMIC Cancer Gene Census.

GENE	Number of sites with increased editing	Number of sites with decreased editing
MDM2	22	0
MDM4	24	15
TP53	0	2
ATM	20	0
VHL	47	0
CASP8	12	2
IDH2	0	11
BRCA2	3	0
ERCC4	2	0

The sites will be considered as significant increased or decreased editing, if at least two tumor types show significant increased or decreased levels, while no more than one tumor type show the significant change in the opposite direction.
