# Supplementary material for: Altered RNA editing in 3′ UTR perturbs microRNA-mediated regulation of oncogenes and tumor-suppressors
Source: Sci Rep. 2016 Mar 16;6:23226. doi: 10.1038/srep23226 (PMC4793219; doi:10.1038/srep23226)
Supplement: Supplementary Information [file srep23226-s1.doc]

# Altered RNA editing in 3’ UTR perturbs microRNA-mediated regulation of oncogenes and tumor-suppressors

Liye Zhang, Chih-Sheng Yang, Xaralabos Varelas, Stefano Monti

**SUPPLEMENTARY METHODS**:

**Data collections**

The accession numbers of dbGaP dataset are phs000235.v2.p1 for lymphoma dataset and phs000467 for neuroblastoma dataset. We only processed the tumor samples with paired DNA-Seq and RNA-Seq data. One of such samples in lymphoma dataset is eliminated due to extremely low coverage in RNA-Seq data. The accession number of the illumina human body map 2.0 is GEO30611. We only used sixteen 100-nt stranded RNA-Seq runs in this study.

**Preprocessing**

Raw FASTQ file for RNA-Seq and paired DNA-Seq sequencing samples were downloaded from dbGaP (Supplementary Table 3). We trimmed the adaptor sequences off the illumina human body map 2.0 by Trim-galore (<http://www.bioinformatics.babraham.ac.uk/projects/trim_galore>, version0.2.8) with adaptor sequence ATCTCGTATGCCGTCTTCTGCTTG (parameters used -s 3 –e 0).

**RNA-Seq alignment**

The RNA-Seq reads were aligned to hg19 reference genome using Tophat v2.0.4 ^1^. The parameters used in Tophat alignment is “--no-coverage-search”. For dbGaP data, the “—library-type” is set to be “fr-unstranded”, while for illumina human body map 100nt stranded RNA-Seq data, the “—library-type” is set to be “fr-firststrand”.

The optical and PCR duplicates were removed using samtools v0.1.18 rmdup function^2^. For the samples with multiple replicates, all bam files were combined to one bam file using samtools merge function.

**DNA-Seq alignment**

The DNA-Seq reads were aligned to hg19 reference genome using bwa v0.5.8 ^3^. Then we performed the standard GATK pipeline (Version 1.6.11) to perform duplicates removal, realignment around indel and recalibrate the quality score ^4^.

**RNA variant calling**

Then mpileup file was generated for each sample and was used to call mutation compared to the reference by using Varscan 2.2.11^5^. The pileup2snp function is used to call the mutation, and p value cutoff was set to 0.01.

**Expression quantification from RNA-Seq data**

We used Cufflinks v2.0.0 to quantify the expression levels of all the transcripts ^6^. We downloaded the transcript annotation files for hg19 from Cufflinks website(<http://cufflinks.cbcb.umd.edu/igenomes.html>). We used the parameters as followed to run Cufflinks: “--compatible-hits-norm --no-update-check --library-type fr-unstranded”.

We performed student’s t-test (unequal sample size and variance) on the expression level of ADAR and ADARB1 to estimate whether they are differently expressed between lymphoma and neuroblastoma.

**Commonly adopted quality filters of RNA editing sites**

The commonly adopted quality filters were applied to the variant calling output to eliminate false positive rate (**Table S2**). These filters are adopted from Varscan manuscript and publications’ recommendations (see Supplementary Table 2 for references). By analyzing the paired DNA-Seq data, the germline and somatic mutations were removed. We only retained the RNA-Seq variant calls where its variant allele has <5% allele frequency in the paired DNA-Seq data.

**RNA editing sites annotation**

The RNA editing sites were searched against the commonly used RNA editing databases ^7, 8^. All RNA editing sites are also annotated by ANNOVAR package ^9^.

**Frequency distribution plot of RNA editing degree**

In the **Figure 2,3** and **Figure S1-3** and **S5**, we compiled the RNA editing sites in all samples. If the same RNA editing sites were identified in multiple samples, they were treated as different data points and not merged.

**Frequency distribution plot of nucleotide substitution type of RNA editing**

In the **Figure 2,3** and **Figure S1-3** and **S5**, we compiled the RNA editing sites in all sample. If the same RNA editing sites were identified in multiple samples, they were treated as one data point. Because the RNASeq data are not stranded RNA-Seq, we were able to be able to infer the sense strand of a pair-end or single-end read. Then we decided to consider both A🡪G and T🡪C as A🡪G type and both C🡪T and G🡪A as C🡪T type.

**Relationship between median levels of RNA editing and the number of under-detected sites in a population**

To generate the box plot of median A-I RNA editing degree for all editing sites, we calculated how many samples with sufficient read coverage (10 used in the current analysis) had no supporting read at all for each editing site. Then we grouped all A-I RNA editing sites based on the number of samples without supporting reads. As the number of RNA editing sites with 5 or more samples with zero supporting read is pretty small (all less than 20), we only plotted the first five groups. For each group, each data point represents the median editing degree of that site among all the samples, the box plots were generated by using Kaleidagraph(version4.1.3).

**Calculation of RNA editing levels in 3’ UTR regions of BT474 and MDA-MB-231 cell lines**

The SRA files were downloaded from NCBI SRA dataset. The SRA files were converted to FASTQ files with tool fastq-dump. Then the alignment to human reference genome hg19 was performed by short read aligner STAR^10^. Then the RNA editing levels for the 3’ UTR region of MDM2 was calculated based on the coverage information extracted by samtools mpileup function^2^. The RNA editing level is calculated as the percentage of G (C) nucleotide among the total of A(T) and G (C) nucleotide at the RNA editing site.

**Calculation of western band signal intensity using ImageJ**

Image is first loaded into ImageJ software and the magnified to 200%. Then the figure will be converted to grey scale, but selecting “Image🡪type->8bit” in the menu. We will subtract background by selecting “Process🡪 subtract Background” with rolling Ball Radius set to 50 and white background. Next, we reverse the image by “Ctrl-Shift-I“ and change the measure scale to pixels by selecting “Analyze🡪Set Scale🡪Pixels”. Lastly, “FreeHand selection” method will be used to measure the area and average intensity for each western blot band. The total signal is calculated by multiply the area and the average signal intensity for each band.

**Details Method of RAAR: RNA-Seq based Alignment Artifact Removal Toolkit**

**Features:**

RAAR consists of two main modules (Sup Figure 1), aimed at the removal of splice-junction artifacts and the handling of multiple alignments, respectively. Together, they aim to remove the majority of the false positive variant calls, and to increase the true positive rate.

## Splice-junction artifact removal

The misalignment of RNA-Seq reads around exon splice-junctions often leads to false positive variant calls, which are considered splice-junction artifacts. Most of the existing approaches to remove splice-junction artifacts rely on the exon-intron boundary annotation of a genome, which work well with species with in-depth genome annotations ^11, 12^. To broaden the applicability of the method to poorly (or non-) annotated species, we developed an algorithm that does not rely on such annotations. Our approach handles the splice-junction removal by directly extracting the splicing junction coordinates from the CIGAR field of RNA-Seq alignment output. If the variant coordinate is within a given distance from predicted splice junction sites, the read will be considered as a false positive supporting read. After removing these supporting reads, if the allele frequency is still above a pre-defined threshold, the variant will not be considered as an artifact.

In addition, our method can handle artifacts caused by sample-specific small deletions, which can be considered as small splicing events. Such artifacts can be removed by performing time-consuming multiple-steps realignment around indels using GATK ^4^. To simplify such process, we first extract the CIGAR field of all variant supporting reads; we then call the small deletions based on the field’s value. Variants that overlap with small deletions are then predicted to be false positive.

## Multiple-alignment artifacts removal

Whole genome duplication events may lead to highly similar regions in the genome. Multiple-alignment artifacts arise from the incorrect alignment of reads to these regions. Removal of Multiple-alignment artifacts is very important, especially in light of the recent finding that all non-canonical (non A-I) RNA editing events reported in a previous study were due to misalignment ^13^. Here we address this problem in two steps.

First, we calculate the percentage of abnormally aligned paired-end reads. These include: a) paired-end reads aligned to different chromosomes; b) reads whose paired-end distance is above the expected length; c) paired-end reads with more than one equally good alignment in the genome. If the percentage of abnormal paired-end reads is above a given threshold, we consider the variant call as a likely false positive.

Next, we perform Blat realignment to resolve incorrect alignments ^14^. For all variant-supporting reads, the best alignment result from Blat is compared with the alignment results from input data (such as Tophat). The reads that map discordantly by Blat and by the input method are considered to be misaligned. The variant is considered to be a false positive, if more than 50% of current variant supporting reads are predicted to be misaligned by Blat.

**Implementation:**

The tools are implemented in three python (2.x) scripts that can be run independently and take standard VCF4.0 format and alignment bam files as input. Generally, these two modules should be run sequentially to obtain the most stringent variant call set.

The deeply sequenced GM12878 cell line in the human ENCODE project has been used to evaluate previous variant calling algorithms ^15^; therefore we decided to test our toolkit on this dataset as well. First, we assessed whether our splice-junction artifact removal module can perform as well as the annotation-based method. The conventional approach removes intronic calls within 4 bases of the intron-exon boundary ^12, 16^. By this criterion, 8299 variants were considered to be false positive. Our approach removed 7687 (93%) without a known annotation file. We performed the same comparison on the Illumina human body map dataset ^17^, which yielded ~90% removal across samples. In addition, our approach identified ~1000 variant calls as false positive due to small deletions in GM12878 dataset.

To test the multiple alignments artifact removal module, we checked whether our method was able to detect known multiple alignment artifacts from published literature ^13^. Indeed, our method removed all 4 validated multi-alignments artifacts correctly (see supplementary material for details).

We have also applied the toolkit to study the DNA mutation and RNA editing in two separate projects. Highly consistent results with previous studies were obtained for both projects (manuscripts in preparation).

In summary, we provide an easy-to-use toolkit to filter alignment artifacts for variant calling from RNA-Seq dataset alone. This toolkit can be easily combined with other tools to identify DNA mutations or RNA editing from RNA-Seq data more accurately.

**SUPPLEMENTARY RESULTS**

## A significant portion of A-I RNA editing sites is edited at low level

The RNA editing call criteria we selected yielded a confident set of RNA editing sites. However, the requirements of a minimum 5% editing level and of a minimum of 5 RNA editing supporting reads likely missed sites with lower RNA editing level and fewer supporting reads. In fact, recent studies report that a low level of editing (~1%) was observed in the majority of *Alu* RNA editing sites ^18^. To address this point, all the RNA editing sites detected based on the above defined stringent requirements were re-examined across all samples to retrieve potentially undetected RNA editing events with lower level of editing or coverage. Since most non-canonical RNA editing sites (any type other than A-I and C-U substitutions) are likely to be false positive ^13^, we focused only on A-I and C-U canonical RNA editing in our datasets.

As an example, the known A-I RNA editing site in the coding region of *AZIN1* manifested low levels of RNA editing (median editing level ~4%) across multiple samples (**Fig S5**). After incorporation of the low-level events retrieved from each dataset, the frequency distribution of all A-I RNA editing events peaked at ~5% editing level in all three cancer types (**Fig 3A and Figs S6**), suggesting that the 5% threshold may have missed highly recurrent but lowly edited A-I sites. However, the median editing level in NB (23%) is still higher than in DLBCL (17%) and HNSC (14%), which is consistent with the results obtained based on the stringent criteria. Noticeably, the editing level of A-I events spanned a very broad range, which is consistent with previous studies ^19^.

## RNA binding proteins may regulate RNA editing level

Our analysis also shows that a preference for increased or decreased editing cannot be solely explained by global *ADAR* gene regulation. Indeed, only decreased RNA editing levels in KICH and KIRP could be explained by a corresponding decreased expression of *ADAR* and *ADARB1* (**Fig S9**). Recent studies suggest that RNA binding proteins may regulate RNA editing levels, for example, *RPS14*, *SRSF9* and *DHX15* repress RNA editing on a site-specific manner ^20, 21^. Therefore, we examined the expression level changes of these three known regulators of RNA editing and tested if the differential expression of these RNA binding genes can explain the preference for increase/decrease in RNA editing. Indeed, the preference for increase/decrease in RNA editing in PRAD, COAD and KIRC could be explained by the expression changes of these genes (**Fig S11**). Expression of these genes increased significantly in COAD and PRAD, thus resulting in decreased RNA editing levels. Conversely, the down-regulation of these genes in KIRC was associated with increased RNA editing levels, despite the decreased expression of *ADAR*.

## Identification of a microRNA-bound RNA editing site that is associated with its mRNA level

We want to examine whether we can identify a RNA editing site that resides on the direct binding site of a gene related to tumor progression and shows significantly different editing levels between tumors and normals. We first extracted the experimentally validated microRNA-mRNA interactions from published results ^22^. We found that several significantly increased RNA editing sites in tumor overlapped with these experimentally validated microRNA-binding 3’ UTR regions. We ranked these “direct microRNA-bound” RNA editing sites by how many tumor types showed an significant increased or decreased editing level compared to paired normals. The most frequent increased RNA editing sites across tumor types resides on the 3’ UTR region of *RNF115*, which promotes tumor growth in breast cancer^23^. As expected, we observe significant and positive correlations between RNA editing and RNF115 mRNA in multiple tumor types including BLCA (correlation R value: 0.38, p value: 0.02), BRCA (correlation R value: 0.44, p value: 1.6e-10), LUAD (correlation R value: 0.50, p value: 0.002). Therefore, this provides support that altered RNA editing in these direct binding regions is associated with elevated mRNA level.

**SUPPLEMENTARY FIGURE LEGENDS**

**Supplementary Figure 1**: Individual sample showed identical patterns to pooled results in lymphoma.

(A-E) Five samples with higher read count in lymphoma dataset were selected. Frequency distribution plots of editing degree and substitution type were generated for each sample. No binning and smoothing was applied.

**Supplementary Figure 2**: Individual sample showed identical patterns to pooled results in neuroblastoma.

(A-E) Five samples with higher read count in neuroblastoma dataset were selected. Frequency distribution plots of editing degree and substitution type were generated for each sample. No binning and smoothing was applied.

**Supplementary Figure 3**: Individual sample showed identical patterns to pooled results in head and neck cancer.

(A-E) Five samples with higher read count in TCGA head and neck cancer dataset were selected. Frequency distribution plots of editing degree and substitution type were generated for each sample.

**Supplementary Figure 4**: The expression level of three ADAR family genes (RPKM).

The box plots of each ADAR gene in each tumor type were generated.

**Supplementary Figure 5**: The editing degrees of an AZIN1 RNA editing site in the samples of lymphoma and neuroblastoma.

The editing degree for each sample was calculated and plotted in the dot plots.

**Supplementary Figure 6**: The majority of A-I RNA sites are edited in a low level.

(A) The frequency distribution plots of editing levels from pooled A-I RNA editing events among all samples in NB after retrieving events with relaxed threshold. (B) The frequency distribution plots of editing level from pooled C-U RNA editing sites among all samples in NB. (C) The frequency distribution plot of variant allele frequency of A-G type somatic mutations in all NB samples. (D) The frequency distribution plot of variant allele frequency of A-G type germline mutations in all NB samples. For all four figures, the bin size is 1 bp, and no smoothing is applied to the data. (E) The frequency distribution plots of editing levels from pooled A-I RNA editing events among all samples in HNSC after retrieving events with relaxed threshold. (F) The frequency distribution plots of editing level from pooled C-U RNA editing sites among all samples in HNSC.

**Supplementary Figure 7**: highly edited A-I RNA editing sites are more frequently observed.

(A-C) The frequency plots of observed RNA editing events were generated for each tumor type. (D-F) The box plot of RNA editing levels for each group ranging from sites observed in all samples to the sites not observed in (n-4) samples (n is the total sample number) were generated for each tumor type.

**Supplementary Figure 8**: The RNA editing sites with more read coverage are more likely to be observed.

(A) For the novel A-I RNA editing sites identified in HNSC, we examined these sites to see how many of these sites were found in the Illumina human body map (n=16). The A-I RNA editing sites colored in red were supported by two or more samples. The A-I RNA editing sites colored by red dots were supported by one sample. The A-I RNA editing sites with no sufficient coverage for all samples were colored by grey. The A-I RNA editing sites colored by blue were not supported by any sample with sufficient coverage. (B-D) For each color group, the boxplot of the read coverage in each site was generated for each tumor type respectively.

**Supplementary Figure 9**: ADAR and ADARB1 gene expression in tumor types with significant increased RNA editing in MDM2 3’ UTR.

The expression data was downloaded by firehose and log2 transformed. The boxplot of the ADAR and ADARB1 genes from paired normal and tumor samples were generated. Student’s t test was applied to test whether the expression between tumors and normals are significant different.

**Supplementary Figure 10**: The IGFBP7 gene expression in tumor types with significant RNA editing changes in coding region.

The expression data was downloaded by firehose and log2 transformed. The boxplot of IGFBP7 from paired normal and tumor samples were generated. Student’s t test was applied to test whether the expression between tumors and normals are significant different.

**Supplementary Figure 11**: The three RNA binding protein gene expressions in three tumor types with significant RNA editing changes.

The expression data was downloaded by firehose and log2 transformed. The boxplot of the DHX15, RPS14 and SRSF9 genes from paired normal and tumor samples were generated. Student’s t test was applied to test whether the expression between tumors and normals are significant different.

**Supplementary Figure 12**: The TP53 gene expression in tumor types with significant RNA editing changes in 3’ UTR region.

The expression data was downloaded by firehose and log2 transformed. The boxplot of TP53 from paired normal and tumor samples were generated. The Student’s t test was applied to test whether the expression between tumors and normals are significant different.

**SUPPLEMENTARY FIGURES**

**Figure S1**


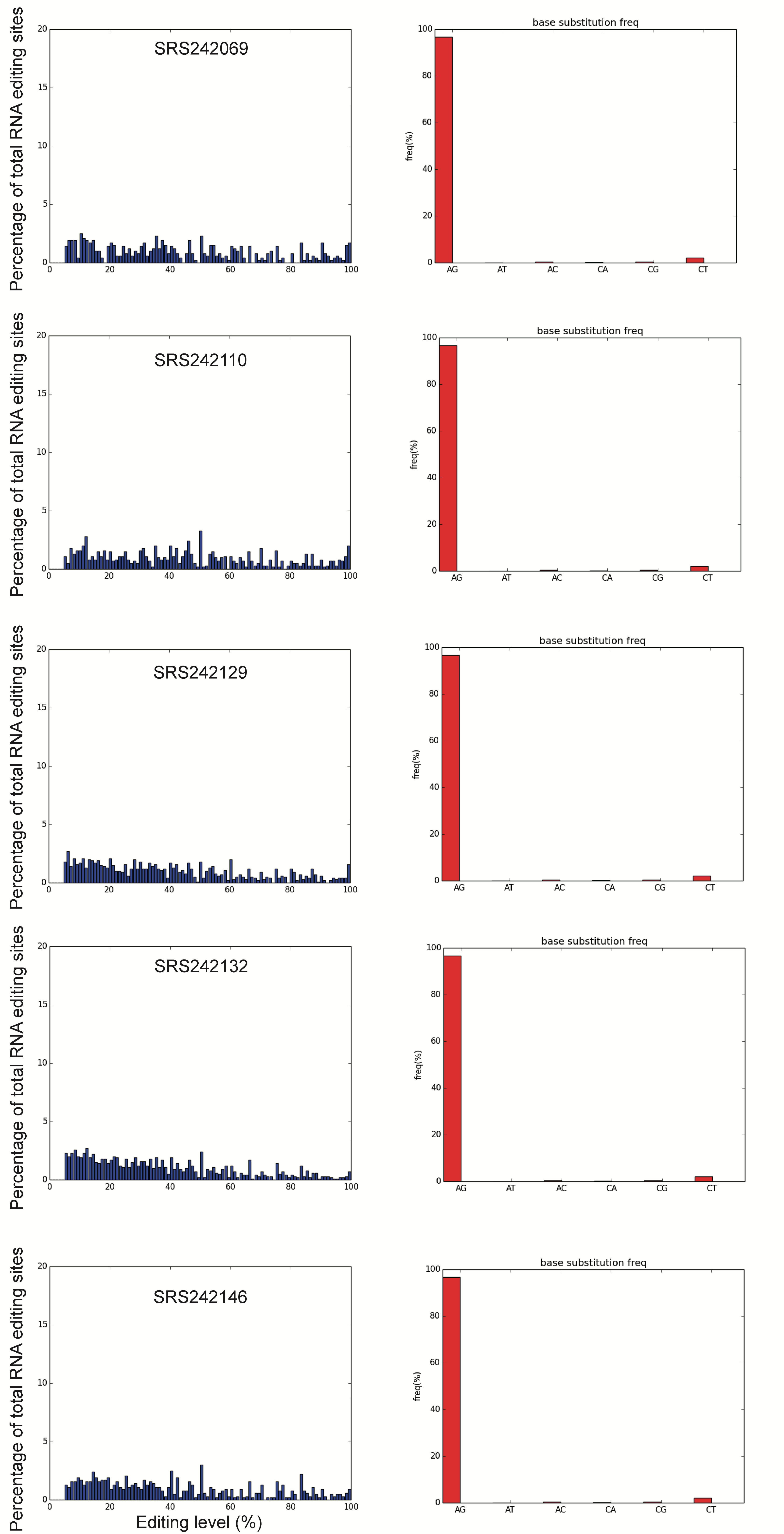


**Figure S2**

**
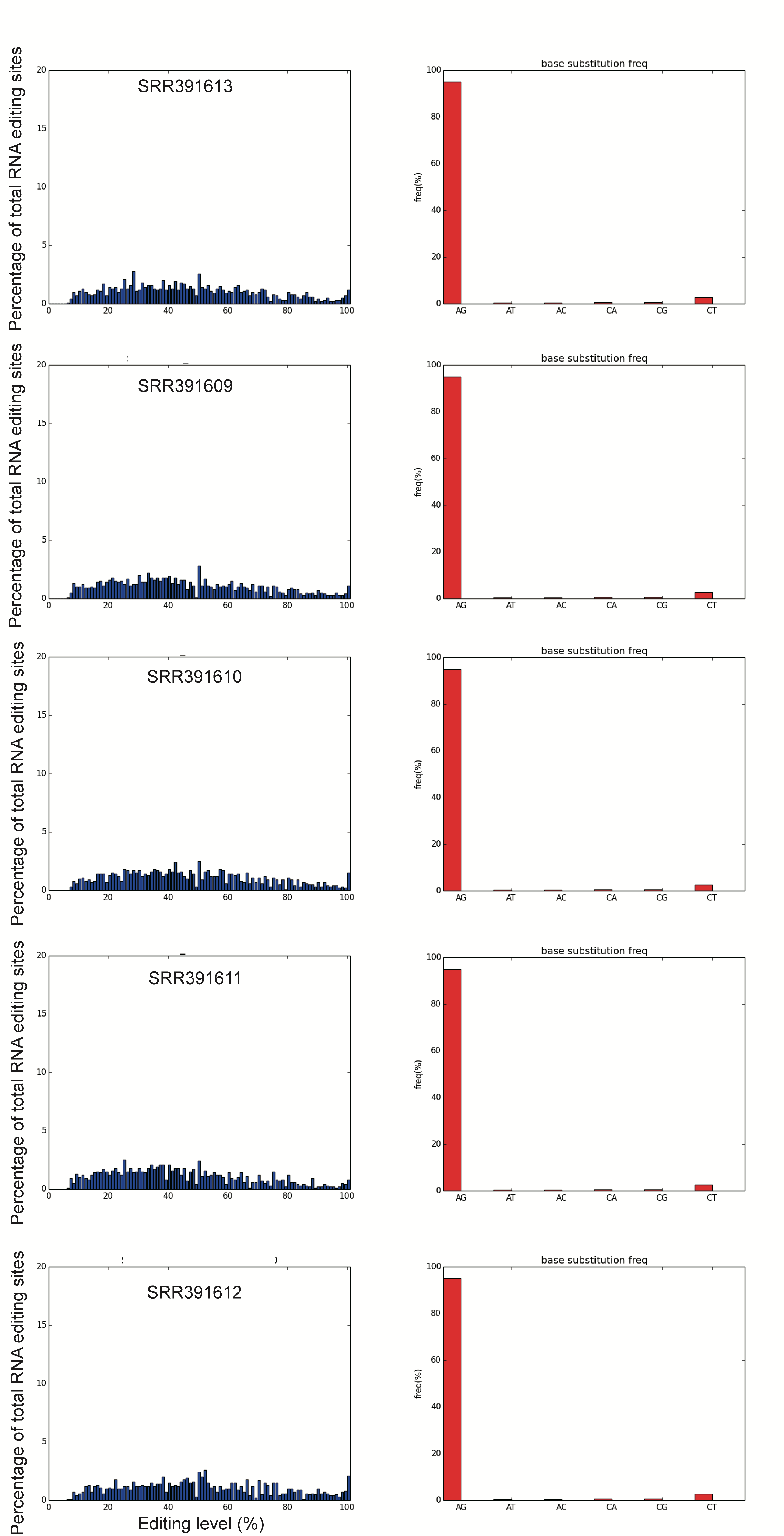
**

**Figure S3**

**
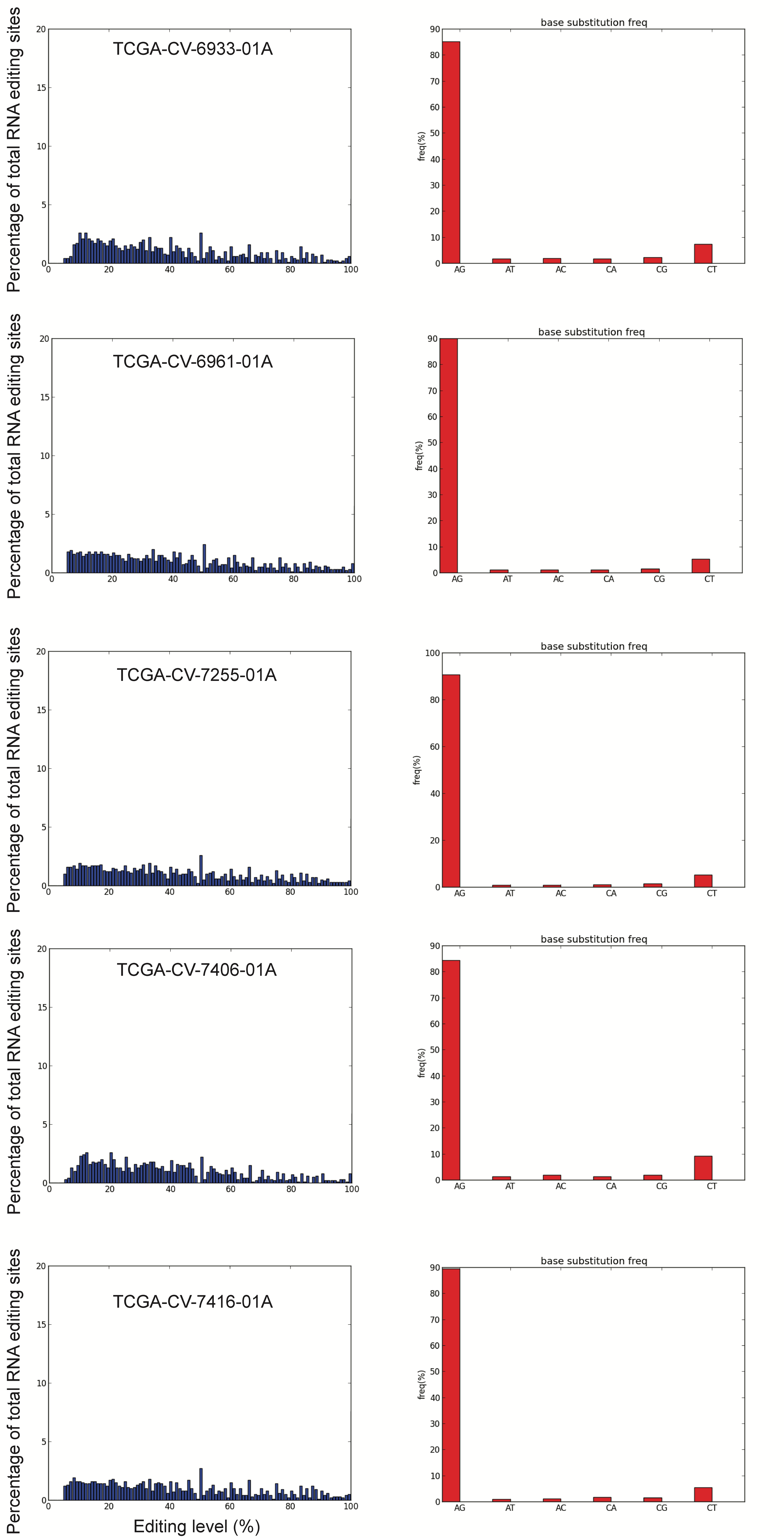
**

**Figure S4**

**
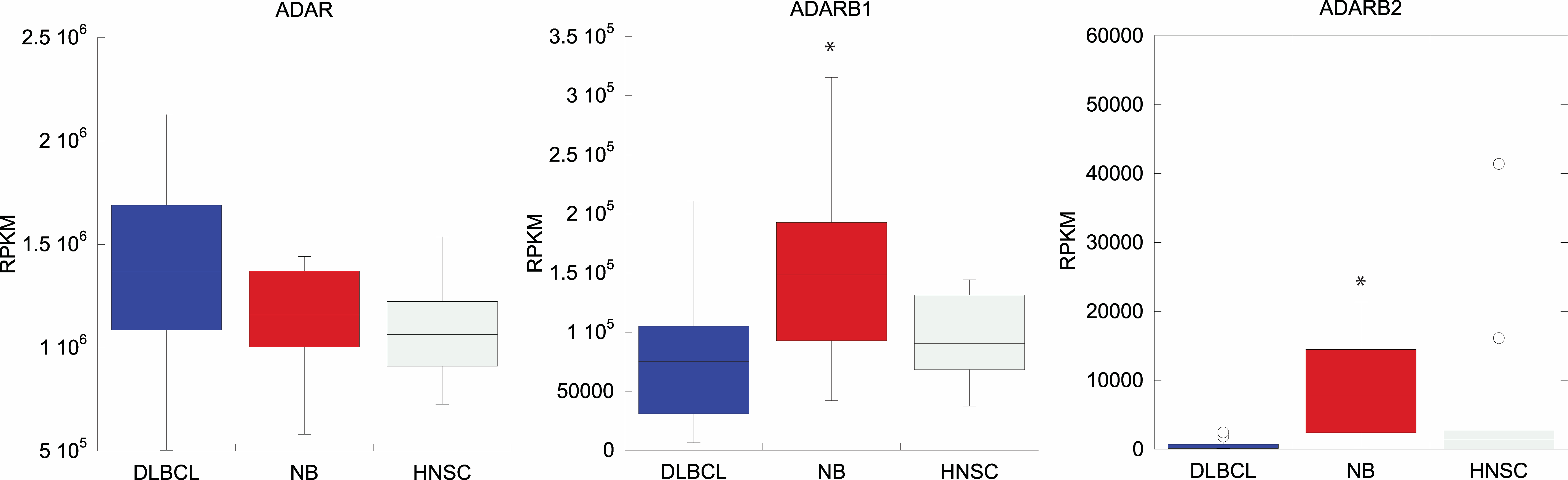
**

**Figure S5**

**
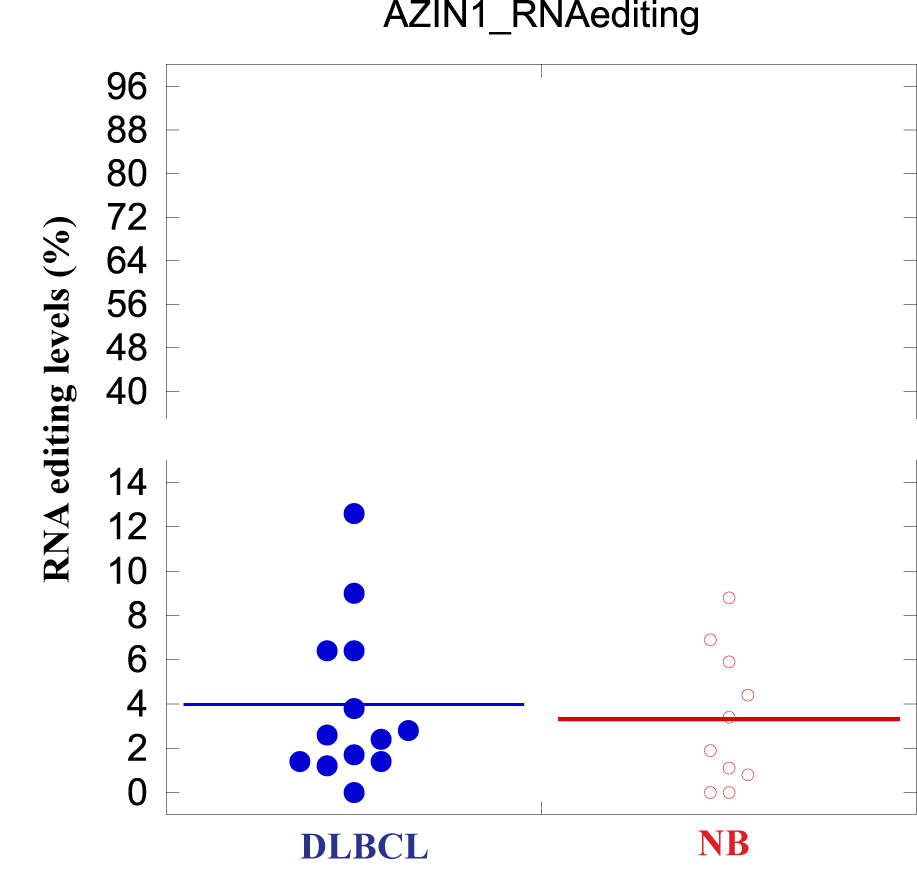
**

**Figure S6**

**
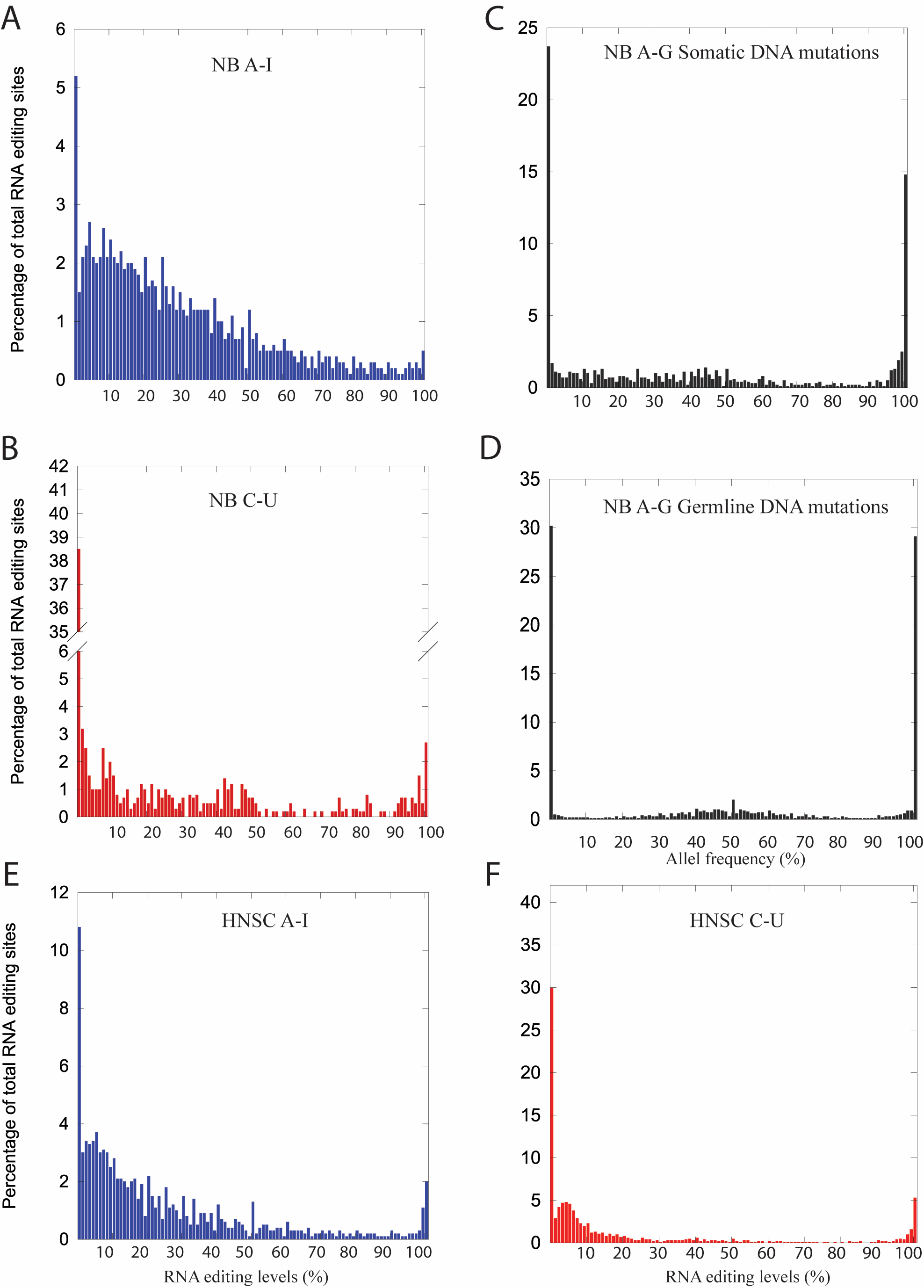
**

**Figure S7**

**
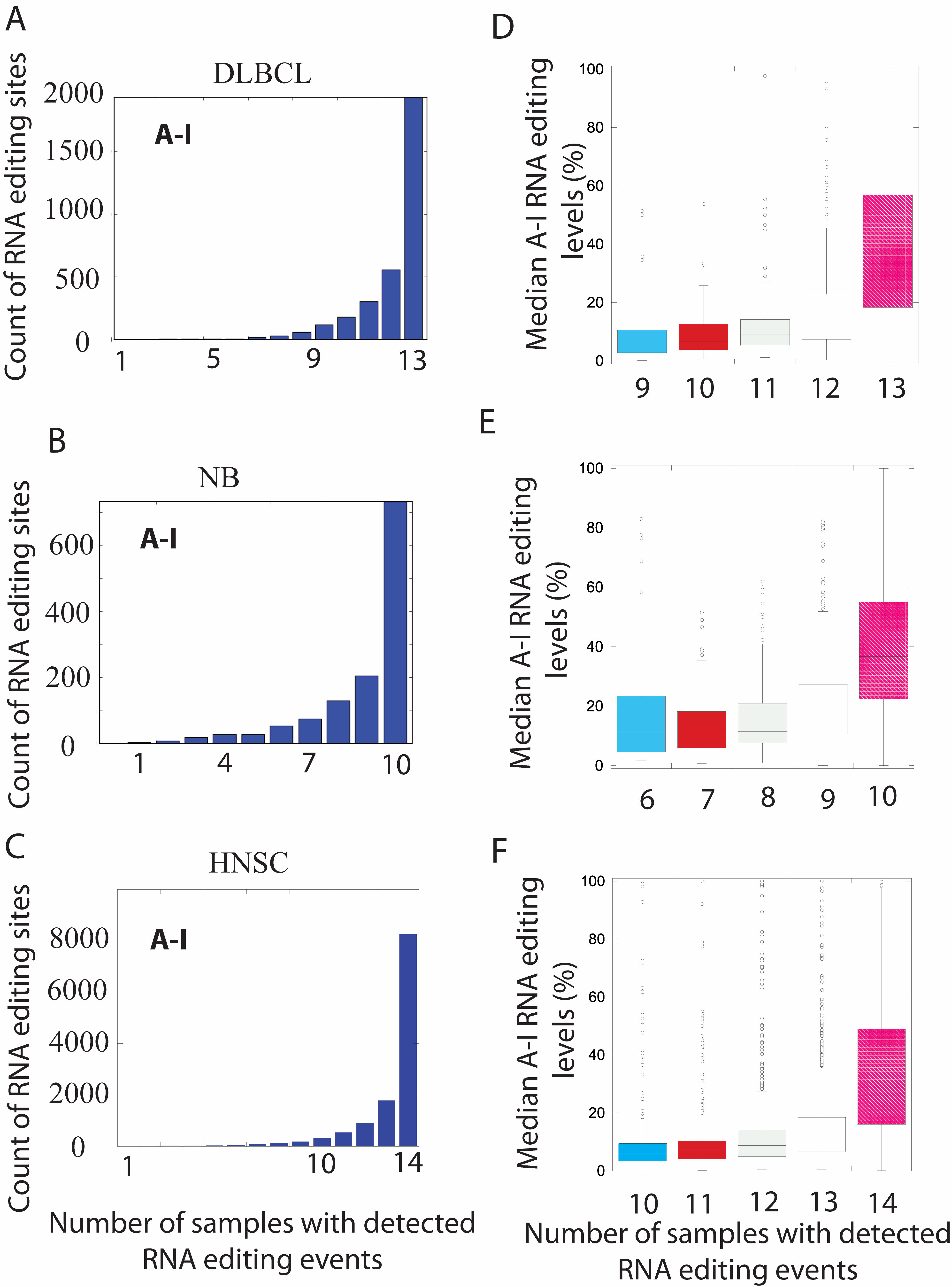
**

**Figure S8**

**
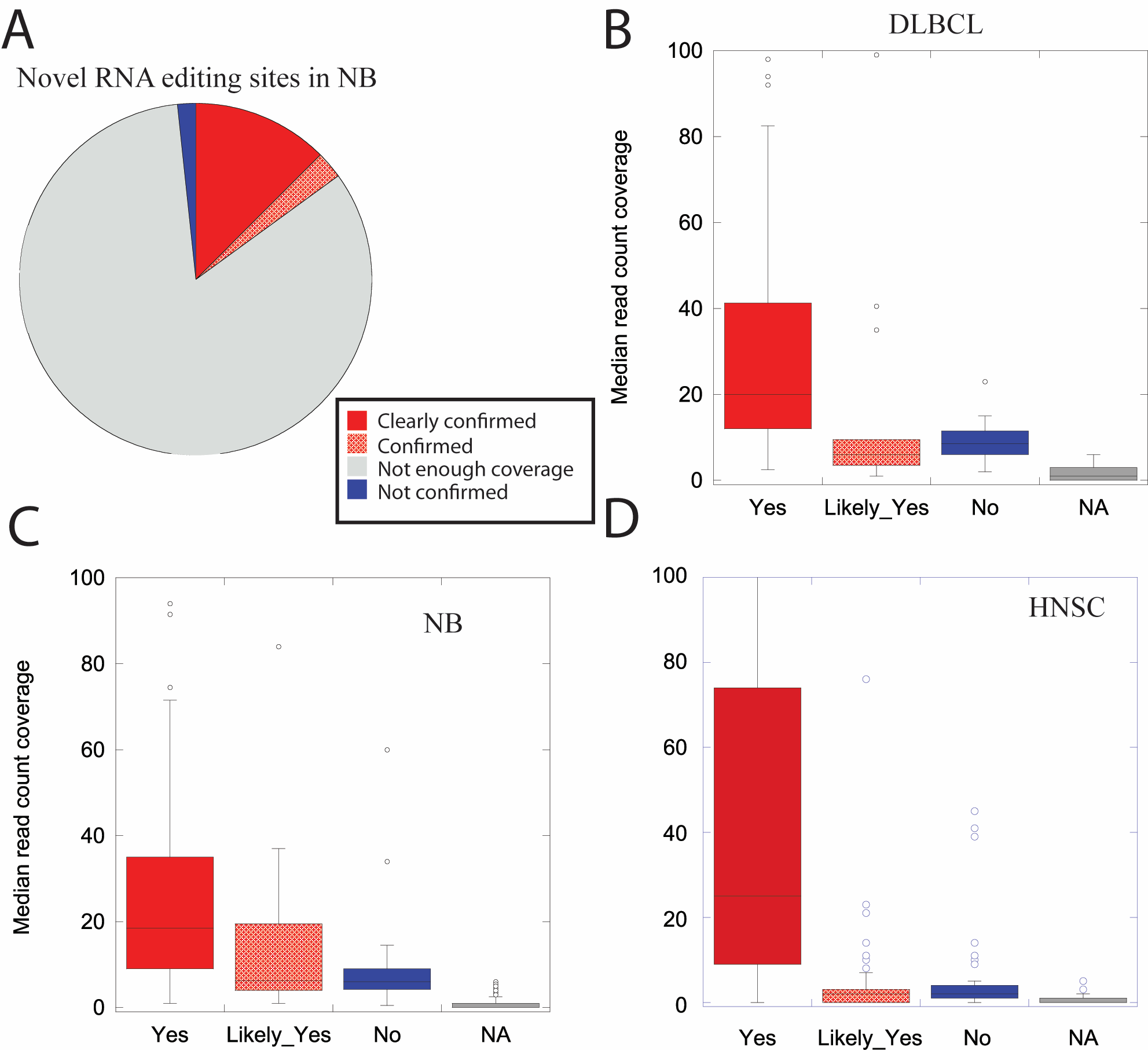
**

**Figure S9**

**
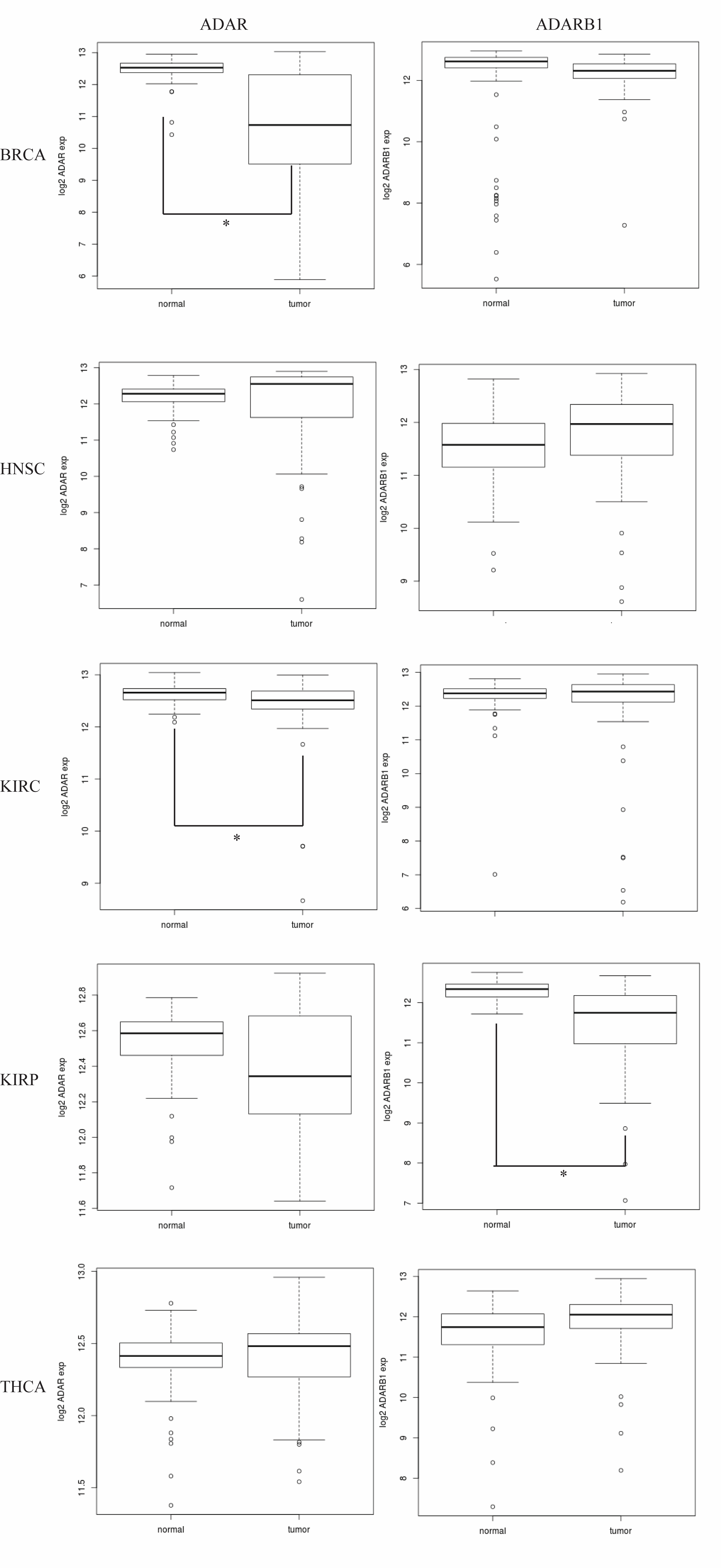
**

**Figure S10**

**
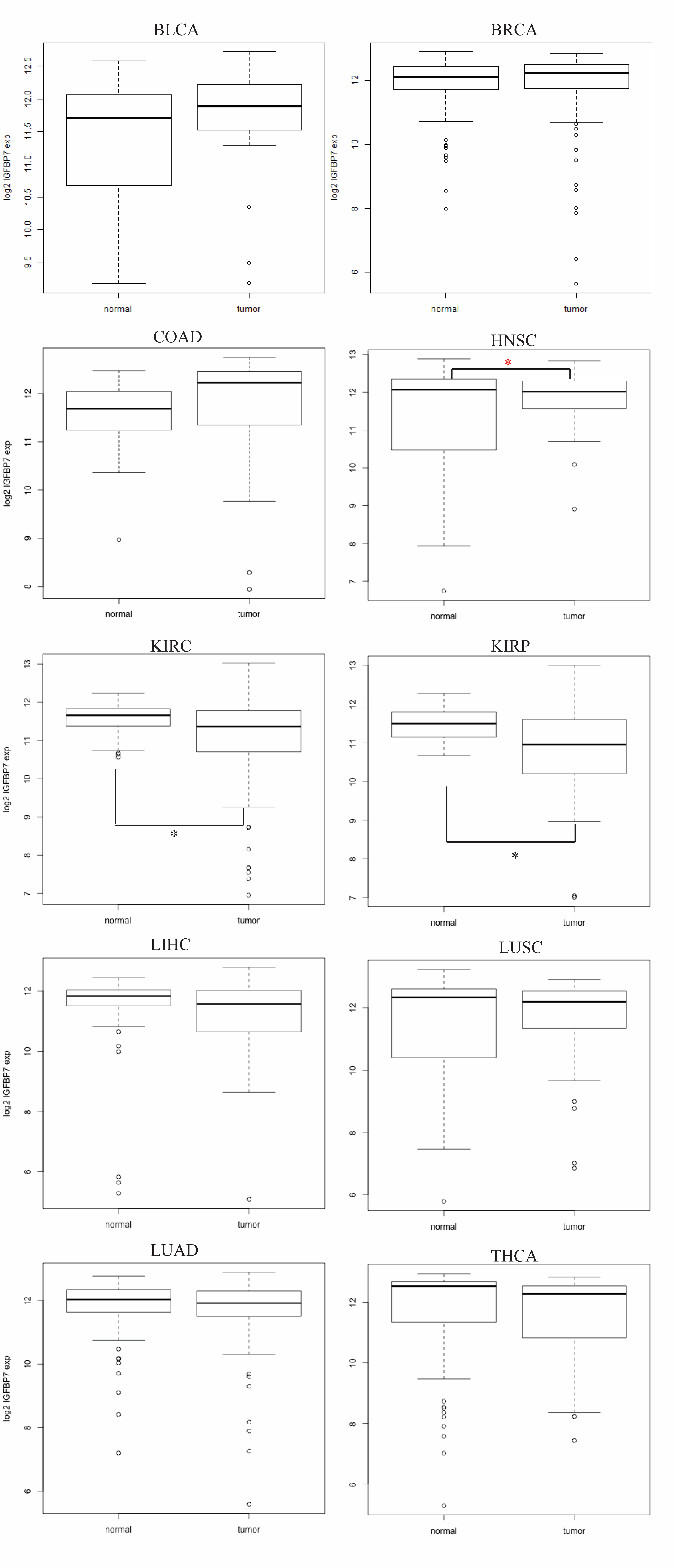
**

**Figure S11**

**
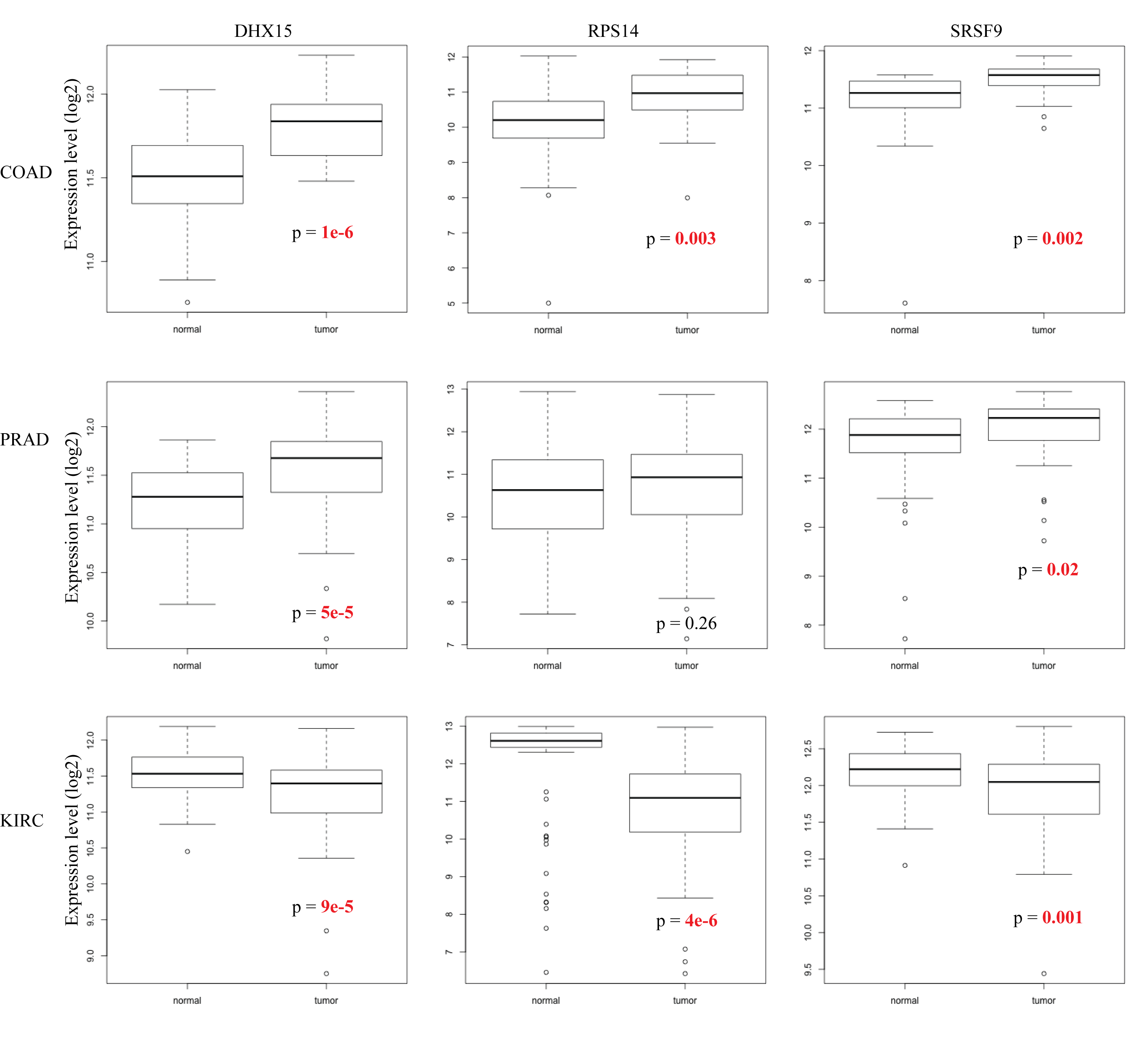
**

**Figure S12**

**
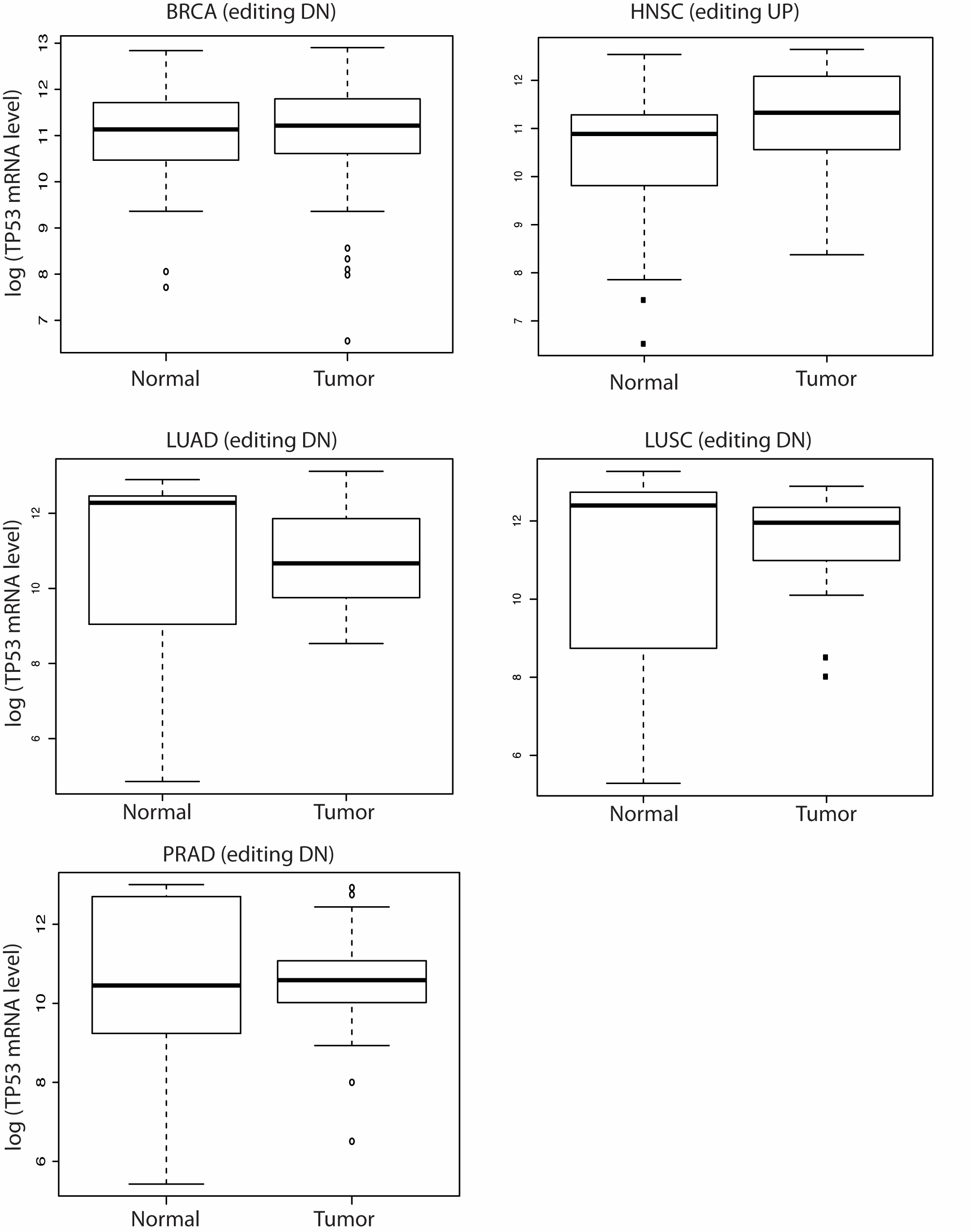
**

**SUPPLEMENTARY TABLES**

**Supplementary Table 1**: Summary of sequencing coverage and number of RNA DNA Difference (RDD) site detected. We use RDD to be more conservative in here, most of the RDD sites are RNA editing sites.

**Supplementary Table 3**: Summary of significant changed RADAR RNA editing sited in the coding region in each tumor type. -1 denotes a significant decreased editing, while 1 denotes a significant increase in editing. 0 denotes no significant change.

**Supplementary Table 4**: Opposite RNA editing changes in the same gene in the different tumor types for all the genes. -1 denotes a significant decreased editing, while 1 denotes a significant increase in editing. 0 denotes no significant change.

**Supplementary Table 2**: Summary of filters applied to obtain high confident RNA editing calls

| Filter Aim | Specific Filter Criteria applied And rationale |
| --- | --- |
| 1. Filter out low frequency events | Allele frequency less than 5% is not considered^5^ |
| 1. Filter out variant calls with strand bias | Any mutations whose strand frequency falls between 1% or 99% will be retained^5^ |
| 1. Filter out variant calls without enough supporting reads | Any mutations with less than 4 supporting reads will be discarded^5^ |
| 1. Filter out variant calls due to misalignment around splice junctions | Filter out intronic calls within 4 bp range to an intron-exon junction^24^ |
|  |  |

The criteria we chose are based on the recommendation from published results.

**Supplementary Table 5**: RNA editing levels at the 3’ UTR region of MDM2

| coordinate | BT474 | MDA-MB-231 |
| --- | --- | --- |
| Chr12_69237004 | 21% | 8% |
| Chr12_69237010 | 60% | 37.5% |

**Supplementary Table 6**: Summary of significantly changed RNA editing sites in each tumor type

|  | **coding** | | **3' UTR** | | **combined** | |
| --- | --- | --- | --- | --- | --- | --- |
| **CANCER_TYPE** | Increased | decreased | Increased | decreased | Increased | decreased |
| **KICH** | 2 | 25 | 186 | 4736 | 188 | 4761 |
| **LUAD** | 0 | 19 | 2105 | 1008 | 2105 | 1027 |
| **PRAD** | 3 | 12 | 288 | 1219 | 291 | 1231 |
| **LUSC** | 20 | 24 | 2299 | 1612 | 2319 | 1636 |
| **LIHC** | 10 | 6 | 929 | 451 | 939 | 457 |
| **BLCA** | 16 | 8 | 1907 | 521 | 1923 | 529 |
| **COAD** | 16 | 6 | 155 | 265 | 171 | 271 |
| **STAD** | 30 | 19 | 3066 | 570 | 3096 | 589 |
| **ESCA** | 15 | 3 | 1633 | 236 | 1648 | 239 |
| **THCA** | 47 | 2 | 4373 | 300 | 4420 | 302 |
| **KIRP** | 58 | 10 | 327 | 1757 | 385 | 1767 |
| **HNSC** | 53 | 2 | 7758 | 52 | 7811 | 54 |
| **KIRC** | 66 | 12 | 5477 | 1255 | 5543 | 1267 |
| **BRCA** | 74 | 18 | 10982 | 590 | 11056 | 608 |

**Supplementary Table 7**: Summary of significantly changed TP53 3’ UTR RNA editing sites in each tumor type. -1 denotes a significant decreased editing, while 1 denotes a significant increase in editing.

| **Editing Coor.** | **chr17_7572004** | **chr17_7571913** | **chr17_7571931** | **chr17_7572026** |
| --- | --- | --- | --- | --- |
| **Annotation** | TP53 | TP53 | TP53 | TP53 |
| **BLCA** | 0 | 0 | 0 | 0 |
| **BRCA** | -1 | 0 | 0 | 0 |
| **COAD** | 0 | 0 | 0 | 0 |
| **ESCA** | 0 | 0 | 0 | 0 |
| **HNSC** | 0 | 0 | 1 | 1 |
| **KICH** | 0 | 0 | 0 | 0 |
| **KIRC** | 0 | 0 | 0 | 0 |
| **KIRP** | 0 | 0 | 0 | 0 |
| **LIHC** | 0 | 0 | 0 | 0 |
| **LUAD** | -1 | -1 | 0 | 0 |
| **LUSC** | -1 | 0 | 0 | 0 |
| **PRAD** | -1 | -1 | 0 | -1 |
| **STAD** | 0 | -1 | 0 | 0 |
| **THCA** | 0 | 0 | 0 | 0 |

**Supplementary Table 8**: Candidate tumor suppressor whose mRNA levels can be affected by RNA editing. The bold and italic tumor types are the tumor types showed a significant positive spearman’s correlation between the RNA editing levels and mRNA levels based on a p value cutoff of 0.05.

| Tumor suppressor genes | Tumor types with significant decreased editing levels | Coordinate of editing site show Pos. correlation | P value of correlation |
| --- | --- | --- | --- |
| AHR | BLCA, KIRP, LUAD, ***LUSC*** | chr7:17384853 | 0.008 |
| CASP8 | BLCA, LUAD, ***LUSC*** | chr2:202152046 | 0.009 |
| KRIT1 | BLCA, BRCA, LUAD, ***PRAD*** | chr7:91829832 | 0.01 |
| PHACTR4 | BLCA, LUAD, LUSC, PRAD,  ***BRCA*** | chr1:28826144 | 0.006 |
| SMYD4 | BLCA, LUAD, LUSC, **BRCA** | chr17:1684044 | 0.001 |

**REFERENCES**

1. Trapnell C, Pachter L, Salzberg SL. TopHat: discovering splice junctions with RNA-Seq. *Bioinformatics* **25**, 1105-1111 (2009).

2. Li H*, et al.* The Sequence Alignment/Map format and SAMtools. *Bioinformatics* **25**, 2078-2079 (2009).

3. Li H, Durbin R. Fast and accurate short read alignment with Burrows-Wheeler transform. *Bioinformatics* **25**, 1754-1760 (2009).

4. DePristo MA*, et al.* A framework for variation discovery and genotyping using next-generation DNA sequencing data. *Nat Genet* **43**, 491-498 (2011).

5. Koboldt DC*, et al.* VarScan 2: somatic mutation and copy number alteration discovery in cancer by exome sequencing. *Genome Res* **22**, 568-576 (2012).

6. Trapnell C*, et al.* Transcript assembly and quantification by RNA-Seq reveals unannotated transcripts and isoform switching during cell differentiation. *Nat Biotechnol* **28**, 511-515 (2010).

7. Kiran A, Baranov PV. DARNED: a DAtabase of RNa EDiting in humans. *Bioinformatics* **26**, 1772-1776 (2010).

8. Ramaswami G, Li JB. RADAR: a rigorously annotated database of A-to-I RNA editing. *Nucleic Acids Res* **42**, D109-113 (2014).

9. Wang K, Li M, Hakonarson H. ANNOVAR: functional annotation of genetic variants from high-throughput sequencing data. *Nucleic Acids Res* **38**, e164 (2010).

10. Dobin A*, et al.* STAR: ultrafast universal RNA-seq aligner. *Bioinformatics* **29**, 15-21 (2013).

11. Picardi E, Pesole G. REDItools: high-throughput RNA editing detection made easy. *Bioinformatics* **29**, 1813-1814 (2013).

12. Piskol R, Ramaswami G, Li JB. Reliable identification of genomic variants from RNA-seq data. *American journal of human genetics* **93**, 641-651 (2013).

13. Piskol R, Peng Z, Wang J, Li JB. Lack of evidence for existence of noncanonical RNA editing. *Nat Biotechnol* **31**, 19-20 (2013).

14. Kent WJ. BLAT--the BLAST-like alignment tool. *Genome Res* **12**, 656-664 (2002).

15. Djebali S*, et al.* Landscape of transcription in human cells. *Nature* **489**, 101-108 (2012).

16. Ramaswami G*, et al.* Identifying RNA editing sites using RNA sequencing data alone. *Nature methods* **10**, 128-132 (2013).

17. Farrell CM*, et al.* Current status and new features of the Consensus Coding Sequence database. *Nucleic Acids Res* **42**, D865-872 (2014).

18. Bazak L*, et al.* A-to-I RNA editing occurs at over a hundred million genomic sites, located in a majority of human genes. *Genome Res*, (2014).

19. Peng Z*, et al.* Comprehensive analysis of RNA-Seq data reveals extensive RNA editing in a human transcriptome. *Nat Biotechnol* **30**, 253-260 (2012).

20. Tariq A, Garncarz W, Handl C, Balik A, Pusch O, Jantsch MF. RNA-interacting proteins act as site-specific repressors of ADAR2-mediated RNA editing and fluctuate upon neuronal stimulation. *Nucleic Acids Res* **41**, 2581-2593 (2013).

21. Washburn MC*, et al.* The dsRBP and inactive editor ADR-1 utilizes dsRNA binding to regulate A-to-I RNA editing across the C. elegans transcriptome. *Cell Rep* **6**, 599-607 (2014).

22. Helwak A, Kudla G, Dudnakova T, Tollervey D. Mapping the human miRNA interactome by CLASH reveals frequent noncanonical binding. *Cell* **153**, 654-665 (2013).

23. Wang Z*, et al.* RNF115/BCA2 E3 ubiquitin ligase promotes breast cancer cell proliferation through targeting p21Waf1/Cip1 for ubiquitin-mediated degradation. *Neoplasia* **15**, 1028-1035 (2013).

24. Ramaswami G, Lin W, Piskol R, Tan MH, Davis C, Li JB. Accurate identification of human Alu and non-Alu RNA editing sites. *Nature methods* **9**, 579-581 (2012).
